# Pubertal timing mediates the association between threat adversity and psychopathology

**DOI:** 10.1017/S003329172400179X

**Published:** 2024-09

**Authors:** Michelle Shaul, Sarah Whittle, Timothy J. Silk, Nandita Vijayakumar

**Affiliations:** 1 School of Psychology, Faculty of Health, Deakin University, Burwood, Victoria, Australia; 2 Melbourne Neuropsychiatry Centre, Department of Psychiatry, The University of Melbourne, Melbourne, Victoria, Australia; 3 Centre for Social and Early Emotional Development, Faculty of Health, School of Psychology, Deakin University, Geelong, Victoria, Australia; 4 Developmental Imaging, Murdoch Children's Research Institute, Parkville, Victoria, Australia; 5 Centre for Adolescent Health, Murdoch Children's Research Institute, Parkville, Victoria, Australia

**Keywords:** adverse childhood experiences, childhood trauma, developmental psychology, puberty

## Abstract

**Background:**

Exposure to adversity in childhood is a risk factor for lifetime mental health problems. Altered pace of biological aging, as measured through pubertal timing, is one potential explanatory pathway for this risk. This study examined whether pubertal timing mediated the association between adversity (threat and deprivation) and adolescent mental health problems (internalizing and externalizing), and whether this was moderated by sex.

**Methods:**

Aims were examined using the Adolescent Brain and Cognitive Development study, a large community sample from the United States. Data were used from three timepoints across the ages of 9–14 years. Latent scores from confirmatory factor analysis operationalized exposure to threat and deprivation. Bayesian mixed-effects regression models tested whether pubertal timing in early adolescence mediated the relationship between adversity exposure and later internalizing and externalizing problems. Sex was examined as a potential moderator of this pathway.

**Results:**

Both threat and deprivation were associated with later internalizing and externalizing symptoms. Threat, but not deprivation, was associated with earlier pubertal timing, which mediated the association of threat with internalizing and externalizing problems. Sex differences were only observed in the direct association between adversity and internalizing problems, but no such differences were present for mediating pathways.

**Conclusions:**

Adversity exposure had similar associations with the pace of biological aging (as indexed by pubertal timing) and mental health problems in males and females. However, the association of adversity on pubertal timing appears to depend on the dimension of adversity experienced, with only threat conferring risk of earlier pubertal timing.

Childhood and adolescence are sensitive periods wherein exposure to adverse and traumatic experiences may disrupt development with enduring ill effects on health and wellbeing (Danese & Baldwin, 2017). Early-life adversity (ELA) includes experiences within the first two decades of life that cause intense or prolonged stress and distress (Danese, 2020). Although there is a well-established association between the experience of ELA and lifetime occurrence of psychopathology (Lewis et al., 2019), the mechanisms through which this risk is engendered remains the subject of investigation. While there is evidence that ELA exposure may have a cumulative effect on mental health (Merrick et al., 2017), there is emerging evidence that different types of adversity exposure have distinct neurobiological sequelae, which uniquely explain detrimental mental health outcomes (Ellis, Figueredo, Brumbach, & Schlomer, 2009; McLaughlin & Sheridan, 2016). One proposed mechanism is differences in the pace of biological development compared to peers, including the timing and pace of pubertal maturation (Colich, Rosen, Williams, & McLaughlin, 2020b).

Two convergent theoretical models, the *threat–deprivation* (McLaughlin & Sheridan, 2016) and *harshness–unpredictability* (Ellis et al., 2009) models, posit that underlying characteristics – or dimensions – of ELAs may explain observed differences in the pace of aging and its relationship with mental health problems. The threat dimension describes adversities characterized by witnessing or experiencing potential or actual harm to bodily integrity, including abuse, domestic, and community violence or serious injury and illness. The deprivation dimension includes exposures characterized by a lack of expected environmental inputs necessary for development, such as low cognitive stimulation, food insecurity, or neglect. Threat and deprivation appear to have distinct effects on the pace of biological aging. Greater exposure to ELAs characterized by threat, but not deprivation, has been associated with accelerated sexual, cellular, and neurological development compared to peers (Colich et al., 2020b). Conversely, deprivation, particularly of energetic resources, has been associated with delayed sexual maturation (Ellis, 2004).

Theoretical explanations for the distinct effects that threat and deprivation carry draw on the harshness-unpredictability model developed from life history theory (Ellis & Del Giudice, 2019). Following this model, changes to the pace of biological development may represent evolutionarily advantageous adaptions based on cues of harshness and unpredictability from the early environment. Harshness cues include the presence of external causes of morbidity or mortality, which may be characterized by threat or deprivation (Ellis, Sheridan, Belsky, & McLaughlin, 2022), and unpredictability cues include the degree of stochastic variability of this harshness exposure. Threat exposures are potential imminent risks to morbidity and mortality, and consequently there may be accelerated sexual maturation in order to increase the probability of successful reproduction in such environments (Brumbach, Figueredo, & Ellis, 2009). Conversely, deprivation exposures that encompass scarcity of bioenergetic resources may delay sexual development as the limited (available) energetic resources are allocated to the maintenance of more critical biological systems (Ellis, 2004). According to life history theory, these evolutionarily adaptive shifts in the pace of sexual maturation are developmental trade-offs that bias shorter-term survival and reproduction. It is theorized that this altered aging pace may leave individuals vulnerable to mental health problems if, for example, other systems do not sufficiently develop (Ellis et al., 2009) or as adaptations to difficult early-life environments subsequently become maladaptive as the environment around the individual changes (Kavanagh & Kahl, 2018).

Pubertal timing reflects an individual's sexual maturation relative to peers of the same age and sex, and is thus able to capture individual differences in certain aspects of biological development that is distinct from chronological age (Mendle, Beltz, Carter, & Dorn, 2019). Off-time pubertal timing, both accelerated and delayed, has been associated with diverse ELA exposures and psychopathology outcomes (Vijayakumar & Whittle, 2023). A meta-analysis by Colich et al. (2020b) found that greater exposure to threat, but not deprivation, was associated with earlier pubertal timing. Notably, sex did moderate the relationship between deprivation and pubertal timing, such that there was a stronger association between deprivation and delayed maturation in samples with more males. This suggests a sex-specific effect of adversity type on pubertal timing. Despite an abundance of literature examining the impact of adversity on puberty, few studies in this area have included males in their sample (Sonuga-Barke, Schlotz, & Rutter, 2010; Sumner, Colich, Uddin, Armstrong, & McLaughlin, 2019), and fewer still have looked at sex differences (Colich et al., 2023; Ho, Buthmann, Chahal, Miller, & Gotlib, 2024; Negriff, Blankson, & Trickett, 2015; Stenson, Michopoulos, Stevens, Powers, & Jovanovic, 2021). While all these studies found sex-specific effects of adversity on pubertal development, the observed direction of altered pacing was inconsistent across adversity types. Accordingly, further research is needed to reconcile these differences.

The predominance of female-only samples in the puberty literature has similarly limited our understanding of potential sex-specific effects in the relationship between pubertal timing and psychopathology. Greater mental health problems have been found for males and females with earlier pubertal timing (Ullsperger & Nikolas, 2017). However, there is some suggestion that females may be more affected by accelerated maturation and are more likely to exhibit internalizing symptoms, while later pubertal timing appears to have greater deleterious effects on males (Graber, 2013).

Moreover, few studies have directly tested whether pubertal timing mediates the effect of dimensional adversity exposure on future mental health, with findings considerably varied. In a study of adolescent females, Colich et al. (2020a) found that earlier age at menarche mediated the association between threat (but not deprivation) exposure and distress, fear, and externalizing disorders. Pubertal timing also mediated the association between threat exposure and mental health in two mixed-sex studies of children, but findings were contradictory. Stenson et al. (2021) found earlier pubertal timing mediated the association between trauma exposure and anxiety in girls alone. Conversely, Colich et al. (2023) found earlier pubertal timing mediated the association between threat and externalizing symptoms in boys alone. Thus, our understanding of pubertal timing as a mediator of the effects of adversity on mental health outcomes, and potential differences across males and females, remains limited.

The current study aims to address these limitations by using a large community sample of male and female adolescents. Specifically, this study examined whether pubertal timing mediates the relationship between childhood experiences of threat and deprivation and mental health problems, and potential sex differences in these associations. It was hypothesized that pubertal timing would partially mediate the relationship between childhood experiences of threat and deprivation and mental health problems, with sex differences in these indirect pathways. Based on the literature, dimension- and sex-specific effects were hypothesized, such that threat exposure would be associated with earlier pubertal timing and deprivation exposure with later pubertal timing. It was also hypothesized that exposure to deprivation may have greater effects on male biological aging and threat may have greater effects on females; however, given the limited literature, the direction of effect (i.e. delayed *v.* accelerated aging) was not specified. Supplementary analyses were also conducted on other complex adversity exposures, specifically household instability (as a measure of environmental unpredictability) and socioeconomic stressors (SESs), as poverty/low socioeconomic status is thought to increase risk of both threat and deprivation experiences (McLaughlin, Weissman, & Bitran, 2019). Study pre-registration is available on Open Science Framework: (https://doi.org/10.17605/OSF.IO/WNRP9).

## Methods

### Participants

Data were drawn from the ongoing longitudinal Adolescent Brain and Cognitive Development (ABCD) study, releases 4.0 and 5.0 (accessed via the National Institute of Mental Health Data Archive's ABCD Collection). ABCD is a community cohort of 11 878 youth recruited from 21 sites across the USA (Garavan et al., 2018). Data for this study were taken from three timepoints: baseline, 2-year follow-up, and 3-year follow-up, spanning ages 9–14 years old. Participants were excluded from the analysis if they were missing baseline data for variables contributing to threat and deprivation adversity measures, site ID, or their recorded sex changed between timepoints. They were also excluded if they had missingness for mental health outcomes at all timepoints (see online Supplementary Fig. S1 for further details). This resulted in a final analytic sample of *n* = 9687 (see Table 1 for sample characteristics). Excluded participants were more likely to be non-Hispanic Black or Hispanic, have lower mean parent education, and have higher baseline mental health problems (online Supplementary Table S1). A subsample who completed baseline data for variables contributing to the SES and household instability adversity measures (*n* = 8003) were used in supplementary analyses.

**Table 1.** Characteristics of the final analytic sample

	Females	Males
*N*	4602	5085
Age (years)		
Baseline	9.92 (0.63) [8.92–11]	9.92 (0.63) [8.92–11.08]
2-year follow-up	12 (0.67) [10.67–13.83]	12 (0.67) [10.58–13.83]
3-year follow-up	12.92 (0.64) [11.42–14.75]	12.92 (0.65) [11.42–14.50]
PDS average		
2-year follow-up	2.4 (0.67) [1–4]	1.9 (0.55) [1–4]
Internalizing problems		
3-year follow-up	5.6 (6.1) [0–49]	4.7 (5.5) [0–44]
Externalizing problems		
3-year follow-up	3.4 (4.9) [0–43]	4.3 (5.8) [0–48]
Race/ethnicity		
Non-Hispanic White	2479 (54%)	2479 (54%)
Non-Hispanic Black	635 (14%)	635 (14%)
Hispanic	882 (19%)	882 (19%)
Non-Hispanic Asian	109 (2%)	109 (2%)
Other	497 (11%)	497 (11%)

*Note.* PDS average, Pubertal Development Scale. Values presented in the following format: frequency/mean (proportion/standard deviation) [minimum, maximum].

### Measures

#### Dimensional adversity exposure

Dimensional adversity models propose that individual ELAs may represent exposure across multiple dimensions. Some ELAs are thought to encompass greater exposure to threat (e.g. abuse) or deprivation (e.g. neglect). However, other ELAs are considered complex exposures that may increase risk for both threat and deprivation experiences, such as low socioeconomic status or poverty (McLaughlin et al., 2019). Furthermore, the experience of multiple ELAs across these dimensions is likely given the high co-occurrence of ELAs and their cumulative effects (Merrick et al., 2017). Consequently, exposure to adversity dimensions in this study was operationalized as continuous latent scores derived from a theory-driven confirmatory factor analysis (CFA). This allowed for dimensional adversity exposure scores that captured the weighted contribution of multiple ELAs and the degree of an individual's exposure.

Sixty-eight items of interest were selected from eight ABCD questionnaires (online Supplementary Table S2) at baseline and were allocated to threat, deprivation, SES, or household instability factors (online Supplementary Table S3). Allocation was as informed by Hoffman et al. (2019) as well as existing studies that have operationalized adversity within dimensional frameworks (Colich et al., 2020a; Sheridan, Shi, Miller, Salhi, & McLaughlin, 2020; Sumner et al., 2019).

#### Pubertal timing

A ‘stage-for-age’ approach was used to calculate pubertal timing scores using the youth-report Puberty Development Scale (PDS) (Petersen, Crockett, Richards, & Boxer, 1988) at 2-year follow-up. Five questions from the PDS were used relating to height, body hair and acne for both sexes; breast growth and menstruation for females; and voice changes and facial hair for males; responses range from 1 – ‘has not begun’ to 4 – ‘seems complete’, and 1 – ‘No’ or 4 – ‘Yes’ for the menarche question. For participants with more than three items missing, mean PDS was imputed (see below for imputation methods). Pubertal timing was operationalized as the residual values from a linear regression of mean PDS on chronological age, calculated separately for males and females (Mendle et al., 2019). Positive and negative values represent earlier and later pubertal timing, respectively.

#### Mental health outcomes

Mental health problems at 3-year follow-up was measured using the caregiver reported Child Behavior Checklist for Ages 6–18 (CBCL), which assesses problems occurring over the past 6 months on a 3-point scale (Achenbach, 2009). Raw summed scores of the internalizing and externalizing problem scales were used in analyses.

#### Covariates

Parent report of children's race and ethnicity at baseline (‘non-Hispanic White’, ‘non-Hispanic Black’, ‘Hispanic’, ‘non-Hispanic Asian’, and ‘Other’) was included in all models (nominal variable with ‘non-Hispanic White’ as the reference), serving as a proxy variable for the additional adversity experienced by minority racial and ethnic groups (Shonkoff, Slopen, & Williams, 2021) and accounting for group-based differences observed in pubertal timing (Mendle et al., 2019) and mental health problems (Lopez et al., 2017). As some participants completed 3-year follow-up after the COVID-19 pandemic started, a binary covariate was included to account for this difference based on the start of the pandemic on March 11, 2020 (World Health Organization) and their interview date. There was *n* = 1355 (47% female) participants with 3-year follow-up data collection prior to the pandemic. Finally, as participants were nested within collection sites and families (for sibling pairs), these group-level identification variables were also included.

### Statistical analyses

All data processing and analyses were conducted in R (see online Supplementary materials for code and specifications of the packages used).

#### Confirmatory factor analysis

CFA was conducted in multiple stages, described in full in the online Supplementary materials. Briefly, data were randomly split into 50% training and 50% testing. First, each of the proposed factors was modeled separately in the training set. Once the models were established in this initial stage, multi-factor CFA modeling (comprising all factors) was undertaken on the training set. Next, the final model was submitted to the hold-out testing set. Once model fit was determined, latent factor scores for threat and deprivation were extracted for participants. Additional latent factor scores for SES and household instability were used in supplementary analyses.

Indices used to assess model fit were χ^2^ goodness-of-fit test, the root mean square error of approximation (RMSEA, a measure of absolute fit that tests the difference between the model and the data per model degrees of freedom), and the comparative fit index (CFI, an indicator of fit compared to the null model). Model fit was deemed adequate if the RMSEA was ⩽0.05, and CFI was ⩾0.95 (Hu & Bentler, 1998). Models were adjusted when they did not meet these criteria. First, items with standardized factor loadings of <0.30 (Tabachnick, Fidell, & Ullman, 2019) were removed. Second, correlations between residuals were added in accordance with modification indices when changes were consistent with theory.

#### Imputations and transformations

There was 2% missingness for 2-year follow-up PDS data and 26% missingness for both internalizing and externalizing problems at 3-year follow-up. There was no missingness for the threat and adversity dimensions as this was an exclusion criterion. Missing values were estimated with multiple imputation using fully conditional specification. Variables used as predictors in the imputation included sex, baseline body mass index, threat and deprivation latent scores as well as age, PDS average, and CBCL internalizing and externalizing raw summed scores from available timepoints (baseline, 1-year, 2-year, and 3-year follow-ups). The number of imputations was set at 10 as the percentage of incomplete cases was 9.45, based on the White, Royston, and Wood's (2010) rule of thumb. Maximum iterations were set to 10 and predictive mean matching was used to calculate missing values. Mean values across the imputed datasets were extracted for each participant and used in subsequent analyses. The pubertal timing variable was *z*-score normalized, threat was log transformed to address positive skew and deprivation was inverted such that positive values corresponded to greater exposure.

#### Mediation

**Figure 1.**
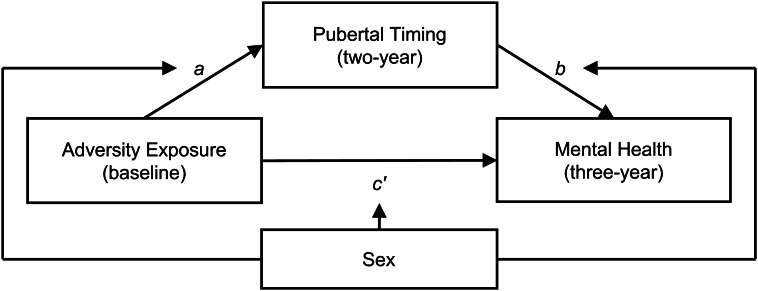
Outline of mediation and moderated mediation models.

Figure 1 outlines the proposed mediation and conditional process models. Potential direct and indirect effects of adversity exposure and pubertal timing on mental health problems were examined through mediation modeling using Bayesian regression analysis. Hierarchical mixed-effects models were run separately for each pairing of the adversity exposure (threat, deprivation) and mental health variables (internalizing, externalizing), with pubertal timing as the proposed mediator. This resulted in four primary models. Each model was estimated using a multivariate approach, wherein two regression equations were simultaneously computed within the Bayesian multilevel model. The regression equations were: (1) mediator ~ independent variable and (2) dependent variable ~ mediator + independent variable. Both included the following covariates: sex, race/ethnicity, and COVID-19 as fixed level one variables, as well as family and site IDs as level two variables with random intercepts (to account for the nested data). As pubertal timing was normally distributed, the first equation was assessed using a Gaussian distribution (online Supplementary Fig. S3). As internalizing and externalizing problems were both positively skewed, the second equation was assessed using a Poisson distribution. Weakly informative priors were used for all models (see online Supplementary materials for further model details and corresponding code). Inferences on indirect mediation effects were based on methods outlined by Yuan and MacKinnon (2009). The indirect (mediation) effect was calculated as the product of the regression coefficients of the *a* and *b* paths using all posterior samples to estimate a distribution. The mean and 95% quantile intervals of posterior distributions were extracted as the effect estimates and credible intervals (CIs) for paths of interest (i.e. the effect estimate had 95% probability of falling within this range based on the posterior distribution). Effects were considered present when the CIs did not include zero. The proportion of variance in mental health problems explained by mediation was quantified as the ratio of the total to the indirect effect (Ditlevsen, Christensen, Lynch, Damsgaard, & Keiding, 2005). Given the multiple models that were examined, we additionally report whether effects remain using 99% CIs (i.e. when 99% CIs do not include zero) as a test of the robustness of findings.

Sensitivity analyses were conducted to assess the specificity of results by adding further covariates to the mediation models. First, to assess whether effects were specific to the type of adversity exposure, the second adversity factor was added (e.g. controlling for deprivation exposure in threat models). Similarly, given that low socioeconomic status represents a risk factor for the predictors and outcomes of interest, a second set of models was run with mean parent education as a covariate. Finally, to assess whether effects were specific to mental health symptoms after the pubertal timing measure (i.e. not simply pre-existing symptoms), the corresponding CBCL problem score at baseline was added in a third set of models.

#### Moderated mediation

Conditional process analysis was used to examine potential sex differences in the hypothesized indirect effect of pubertal timing in adversity exposure and mental health problems (online Supplementary Fig. S5). The four primary mediation models described above were adjusted to include an interaction term of sex on all pathways and rerun. Distributions for the conditional effects were calculated using a product of coefficients method with the equations outlined by Hayes and Rockwood (2019). Corresponding code is available in the online Supplementary materials. The presence of conditional indirect effects was assessed based on the 95% CIs as described above.

## Results

### Confirmatory factor analysis

For the full sample, Bartlett's test of sphericity χ^2^(2145) = 230 138.70, *p* < 0.001 and the Kaiser–Meyer–Olkin (KMO) measure of sampling adequacy (KMO = 0.89) found sufficient correlation and shared variability among the items, indicating that factor analysis was appropriate.

Modifications to items included in each factor at each stage of the CFA are outlined in online Supplementary materials. The final two-factor model of threat and deprivation had adequate fit: χ^2^(654, *n* = 10 964) = 10 520.87, *p* < 0.001, RMSEA [90% CI] = 0.037 [0.036–0.038], CFI = 0.978. Item loadings for this model are presented in Table 2.

**Table 2.** Factor loadings for adversity items in final two-factor model

Factor	Item description	Two-factor		
		Unstd.	s.e.	Std.
Threat	Beaten to the point of having bruises by a family member	1.00	–	0.86
	Death threat by non-family member	1.01	0.02	0.87
	Death threat by family member	1.06	0.03	0.91
	Sexual abuse by a family member	1.11	0.03	0.96
	Sexual abuse by a non-family member	1.04	0.02	0.89
	Sexual abuse by a peer	0.89	0.02	0.76
	Witnessed domestic violence	0.73	0.03	0.63
	Witnessed act of terrorism	1.14	0.03	0.98
	Witnessed death or destruction in war zone	1.09	0.03	0.94
	Witnessed shooting or stabbing in community	0.96	0.02	0.82
	Family members throwing objects in anger	0.41	0.04	0.36
	Experienced bullying	0.36	0.04	0.31
	Experienced serious car accident	0.57	0.03	0.49
	Witnessed/experienced fire causing damage/injury	0.72	0.03	0.62
	Witnessed/experienced natural disaster causing damage/injury	0.70	0.03	0.61
	Learned of sudden/unexpected death of loved one	0.42	0.03	0.36
Deprivation	Makes me feel better after talking over my worries with him/her (primary)	1.00	–	0.71
	Makes me feel better after talking over my worries with him/her (secondary)	0.53	0.02	0.38
	Smiles at me very often (primary parent)	0.91	0.02	0.64
	Smiles at me very often (other caregiver)	0.54	0.02	0.39
	Is able to make me feel better when I am upset (primary parent)	1.05	0.02	0.74
	Is able to make me feel better when I am upset (other caregiver)	0.58	0.02	0.41
	Believes in showing his/her love for me (primary parent)	1.07	0.02	0.76
	Believes in showing his/her love for me (other caregiver)	0.54	0.02	0.38
	Is easy to talk to (primary parent)	0.91	0.02	0.64
	Is easy to talk to (other caregiver)	0.50	0.02	0.35
	We fight a lot in our family [*r*]	−0.78	0.02	−0.55
	Family members rarely become openly angry	−0.57	0.02	−0.4
	Family members hardly ever lose their tempers	−0.63	0.02	−0.45
	Family members often criticize each other	−0.69	0.02	−0.49
	If there is a disagreement in our family, we try hard to smooth things over and keep the peace [*r*]	−0.71	0.03	−0.50
	Family members often try to one-up or outdo each other [*r*]	−0.52	0.02	−0.37
	In our family, we believe you don't ever get anywhere by raising your voice	−0.47	0.02	−0.33
	How often do your parents/guardians know where you are?	0.62	0.02	0.44
	How often do your parents know who you are with when you are not at school and away from home?	0.55	0.02	0.39
	If you are at home when your parents or guardians are not, how often do you know how to get in touch with them?	0.47	0.02	0.33
	How often do you talk to your parent or guardian about your plans for the coming day, such as your plans about what will happen at school or what you are going to do with friends?	0.52	0.02	0.37
	In an average week, how many times do you and your parents/guardians, eat dinner together?	0.46	0.02	0.33

*Note.* Unstd., unstandardized estimate; s.e., standard error; Std., standardized estimate; SES, socioeconomic stressors; *r*, reverse scored item.

### Mediation models

**Figure 2.**
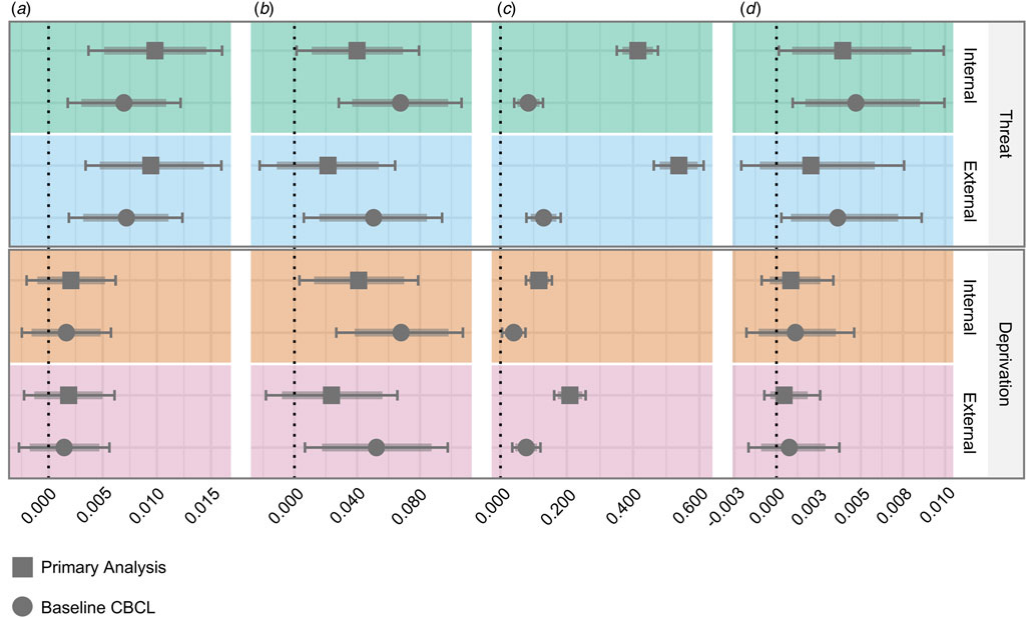
Indirect effects of pubertal timing on adversity and mental health problems. *Note.* Bayesian mixed-effects regression models were used to estimate associations between adversity dimensions (threat and deprivation), pubertal timing, and mental health problems (internalizing and externalizing). Points represent the mean quantile interval of posterior distribution estimates, with values labeled on the bottom horizontal axis. The 99% CIs are represented with capped error bars and 95% CIs with an overlapping bolded line. Mediation model pathways are shown in alphabetized vertical panels: (*a*) adversity exposure and pubertal timing, (*b*) pubertal timing and mental health problems, (*c*) *adversity* exposure and mental health problems, and (*d*) indirect effect of pubertal timing. Models are presented in horizontal panels and are labeled on the right vertical axis. The results were unchanged when mean parent education and the additional adversity measure were included as covariates (results are available in online Supplementary Table S9).

Correlations of adversity dimensions, pubertal timing, and mental health problems are presented in online Supplementary Table S8. Results of the mediation analyses are presented in Fig. 2 and online Supplementary Tables S9 and S10. As illustrated, higher levels of exposure to threat and deprivation were associated with more internalizing and externalizing problems. Greater exposure to threat was also associated with earlier pubertal timing, but no such associations were identified for deprivation.

Earlier pubertal timing was associated with greater internalizing problems, and it also mediated the association between threat and internalizing problems (both with and without controlling for baseline problems). Pubertal timing explained ~1% of the variance in the effect of threat on internalizing problems in these models (online Supplementary Table S9). While earlier pubertal timing was also associated with greater externalizing problems, it only mediated the relationship between threat and externalizing problems when controlling for baseline problems. Pubertal timing accounted for 2.8% of the explained variance in externalizing problems in this model. There were no indirect effects between deprivation and internalizing or externalizing problems via pubertal timing.

The above results were consistent when the more stringent criterion was applied (i.e. 99% CIs did not contain zero). Further, the results remained consistent when controlling for parent education or the second adversity exposure (online Supplementary Table S9).

### Sex differences in the mediation of pubertal timing

**Figure 3.**
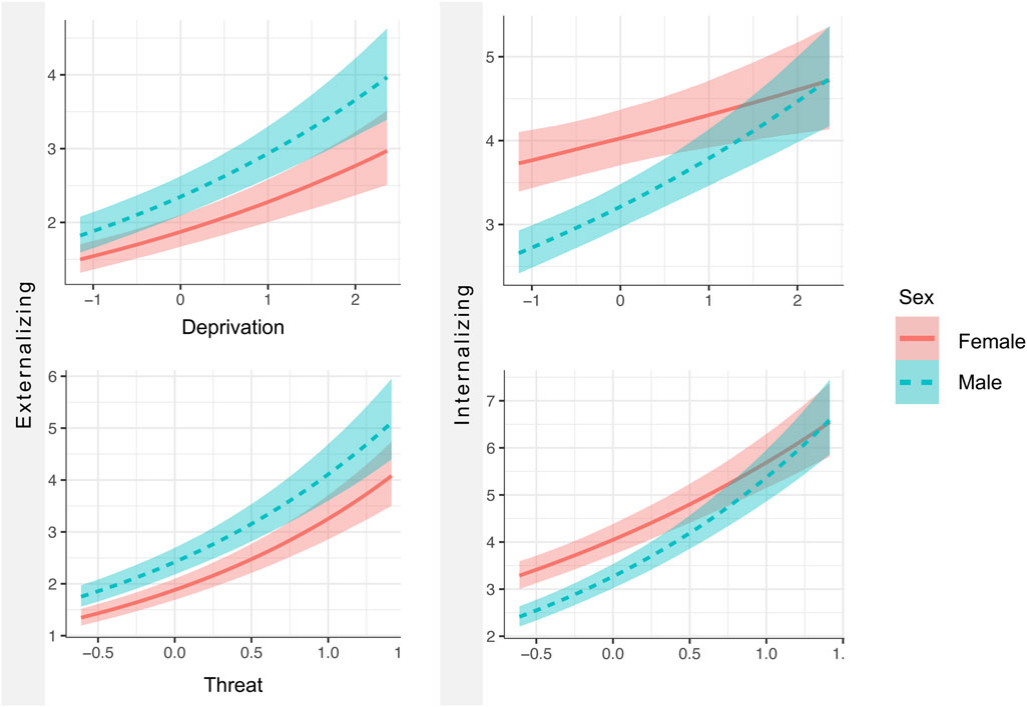
Sex differences in the direct effect of adversity exposure on mental health problems. *Note.* Plotted data are the posterior distribution of a Bayesian regression analysis. Dimensional adversity exposure is plotted on the *x*-axis and mental health problems on the *y*-axis. Effects for females represented in solid line and males in dotted line. The effect of adversity on internalizing problems was significant at 95% CIs for males (threat [0.14350–0.48423], deprivation [0.08053–0.31319]) and females (threat [0.07175–0.24211], deprivation [0.04026–0.15660]).

Results of the moderated mediation are presented in the online Supplementary materials (Table S11). Sex differences were identified in the associations of threat and deprivation on internalizing problems, which were stronger in males. Specifically, while both sexes had similar levels of internalizing problems at high levels of adversity, females had greater problems than males at low levels of adversity (Fig. 3). These associations remained significant in both sexes when examined separately. However, moderated mediation models failed to identify any sex differences in the indirect effect of adversity on mental health problems via pubertal timing.

Post-hoc exploratory analysis was conducted to further examine potential sex differences of deprivation exposure on pubertal timing. As seen in online Supplementary Fig. S6, at greater levels of deprivation exposure, males showed more delayed pubertal timing while females showed earlier timing. Linear regression found this sex difference to be non-significant, however (*b* = −0.057, s.e. = 0.032, *t*(9582) = −1.805, *p* = 0.071).

### Supplementary analyses

Supplementary models (employing the same methods as the primary mediation models) found that greater exposure to both SES and household instability were associated with earlier pubertal timing and greater internalizing and externalizing problems (see online Supplementary Table S12). Indirect effects of both SES and household instability via pubertal timing were found for internalizing, but not externalizing, problems.

## Discussion

This longitudinal study examined whether pubertal timing, as a marker of biological maturation, mediates the relationship between adversity exposure and mental health problems during early adolescence. We found that earlier pubertal timing (defined as pubertal stage relative to same-age and -sex peers) partially mediated the relationship between adversity and mental health problems when the exposure was characterized by actual or threatened physical or sexual harm. Mediation was present in both male and female adolescents. Notably, the indirect effect was only present for externalizing problems when controlling for baseline problems, suggesting that mediation was specific to change in externalizing problems over late childhood to early adolescence. No such indirect effects were found for adversity exposure characterized by physical and emotional deprivation. While there were sex differences in the relationship between adversity exposure and internalizing problems, sex did not moderate indirect effects between adversity exposure and mental health problems via pubertal timing.

We found that greater exposure to adversity in childhood and earlier pubertal timing were both associated with higher levels of internalizing and externalizing problems in 13–14 year olds. The association of adversity and mental health problems remained when controlling for earlier mental health problems at 9–10 years of age. This is consistent with prior findings that childhood adversity exposure is associated with increasing mental health symptoms over the course of childhood and adolescence (Nivard et al., 2017). Although present in both males and females, a sex difference was evident. While females had greater levels of internalizing problems at low levels of adversity (reflective of the higher prevalence of these symptoms in female adolescents [Gutman & Codiroli McMaster, 2020]), no difference was observed at higher levels of adversity. This suggests a similar susceptibility to symptoms in both sexes following exposure to childhood adversities. The significant associations of earlier pubertal timing on mental health problems in both sexes are consistent with the findings of a prior meta-analysis by Ullsperger and Nikolas (2017).

The current findings also contribute to theoretical understandings of the relationship between adversity and psychopathology, providing support for dimensional models that propose explanatory mechanisms are specific to the type of adversity experienced (Ellis et al., 2022). Specifically, we found earlier pubertal timing (at approximately 12 years of age) following childhood adversities characterized by threat but not deprivation. Moreover, we found that earlier pubertal timing partially explained the relationship between threat exposure and later mental health problems. This study extends the existing literature by identifying this mediating effect in both males and females within a large, community sample using longitudinal data. It is consistent with previous studies that have found markers of accelerated biological aging across reproductive, cellular, and neural systems following exposure to threat in early life (Colich et al., 2020b). From an evolutionary perspective, it may have been more advantageous for individuals within a high mortality-risk environment to prioritize development of adult-like capacities, such as sexual maturation, to increase the likelihood of successful reproduction (Ellis et al., 2009). Thus, a faster pace of development may represent an adaptive response to an early environment with greater mortality threat cues. While the precise mechanisms that trigger accelerated aging following threat are not well understood, acceleration does appear to occur across multiple biological systems (Colich et al., 2020b). Earlier pubertal timing has been associated with more progressed DNA methylation (Sumner et al., 2019) and a more ‘adult-like’ brain (Gur et al., 2019), including reduced functional connectivity within cortico-limbic regions (Vijayakumar, Whittle, & Silk, 2023).

The finding that pubertal timing mediated both internalizing and externalizing problems supports proposals that accelerated biological aging poses a transdiagnostic risk factor for psychopathology following childhood trauma (McLaughlin, Colich, Rodman, & Weissman, 2020). A faster aging strategy may pose a trade-off at the expense of more complex neuropsychological development (Colich et al., 2020b), which may be compounded by greater exposure to social circumstances that are incongruent with adolescents' social-emotional maturation. For example, early-maturing adolescents are more sensitive to negative interpersonal environments, with peer relationships in particular moderating the impact of early-pubertal development on mental health problems (Vijayakumar & Whittle, 2023).

Contrary to hypotheses, we failed to identify any associations between childhood exposure to deprivation-related adversities and pubertal timing, even when controlling for co-occurring threat exposure. This is inconsistent with prior findings by Sumner et al. (2019), who identified delayed pubertal timing in those exposed to deprivation. We speculate that differences may be driven by characteristics of this study, including the age range of our sample (10.5–13.5 years) that was too young to capture the effects of delayed timing, which becomes more evident in mid-adolescence. Comparably, a previous study with a sample of similar age also failed to identify any associations between deprivation and pubertal timing in either sex (Colich et al., 2023). Furthermore, our operationalization of deprivation comprised of psychosocial forms of neglect, including primary caregivers' emotional warmth and responsiveness as well as supervision. Overall, the literature suggests distinct effects on biological aging for deprivation that is bioenergetic *v.* psychosocial, which is consistent with the findings of this study. Previous studies examining severe material deprivation and food insecurity in childhood (Ellis et al., 2009; Kyweluk, Georgiev, Borja, Gettler, & Kuzawa, 2018) have more consistently found delayed pubertal timing than those examining psychosocial neglect (Colich et al., 2020b). Accordingly, the relationship between psychosocial deprivation and mental health problems may be better explained through other pathways, such as poorer executive functioning (Miller et al., 2018; Miller, Machlin, McLaughlin, & Sheridan, 2021; Schafer et al., 2022). In the current study, material deprivation was captured distally by the SES factor included in the supplementary analysis. Earlier pubertal timing was associated with both greater socioeconomic disadvantage and greater variability in household structure, and partially explained their association with later internalizing symptoms. This provides support for approaches distinguishing socioeconomic indicators from deprivation (Colich et al., 2020b) (especially psychosocial neglect), although the inconsistent direction of associations compared to prior literature warrants further investigation.

While we failed to identify hypothesized sex differences in pubertal timing pathways, we are cautious to interpret this as support for a lack of sex differences in pubertal timing following deprivation, especially given the limitations of our younger sample. This represents an area in need of further exploration with a broader age range that can better capture delayed pubertal timing.

### Limitations and future directions

Several limitations warrant further discussion to contextualize current findings. First, we used a common/covariance factor approach to operationalize dimensional adversity exposure. While this is consistent with several recent studies that have used CFA to operationalize adversity dimensions (Miller et al., 2021; Usacheva, Choe, Liu, Timmer, & Belsky, 2022), there is concern that this approach may not fully capture exposure (McLaughlin, Weissman, & Flournoy, 2023). Other frequently used approaches to operationalizing adversity exposure similarly have their limitations and a best-practice measure is yet to be established (McLaughlin, Sheridan, Humphreys, Belsky, & Ellis, 2021). Newer composite-based factor approaches may overcome such issues and represents important avenues for future research (McLaughlin et al., 2023).

Second, we operationalized ELA as events occurring at or before baseline measurements. As some participants had already begun puberty by baseline, it is possible that some adversity exposures occurred following puberty onset. This does limit our ability to make causal inferences or to examine the impact of adversity during specific windows of development. Very few studies have examined pubertal timing following pre-pubertal adversity exposure (Ellis & Garber, 2000), or controlled for adversity timing. Given that there is some evidence that the first few years of life may be a sensitive window for the impact of adversity on biological aging (Marini et al., 2020), this is an area that warrants further research.

Third, this study's findings had relatively small effect sizes (most lower bounds of the CIs sitting close to zero), which is a described phenomenon of the ABCD study (Owens et al., 2021). Notably, all effects in the primary analysis remained even at 99% CI. Additionally, the mediation effects found in this study accounted for between ~1% and 3% of the total effect. The small proportion of the relationship between threat and mental health problems mediated by pubertal timing suggests that there are likely other factors influencing this relationship. This aligns with a developmental psychopathology perspective that mental health outcomes are equifinal (i.e. diverse pathways can lead to the same outcome), and that similar life experiences can lead to heterogenous outcomes between individuals (multifinality) (Cicchetti & Rogosch, 1996). Future research could compare effect sizes of other potential mediators of this relationship, such as other measures of biological aging, social-emotional processing, or cognitive functioning (McLaughlin et al., 2020; Miller et al., 2018).

Fourth, this study should be replicated using youth report of mental health symptoms given observed discrepancies between adolescent and parent reports (Barch et al., 2018).

Finally, this study used a community sample from the USA. While broadly representative of the American Community Survey, the ABCD sample overrepresents individuals from higher-educated and higher-income families (Karcher & Barch, 2021). Furthermore, a pattern of selective attrition was observed in this study. Participants excluded due to missingness had higher baseline mental health symptoms, adversity exposure, and minority racial status. Accordingly, generalizability of findings to the broader US population (and other Western countries with similar demographic stratification) may be impacted (Kyweluk et al., 2018). There are likely important effects of intersecting sociodemographic and genetic factors that were not explored in this study. For example, some sociodemographic groups are more likely to experience trauma and adversity (Slopen et al., 2016), while minoritized racial groups also experience the additional adversity of ongoing structural and interpersonal discrimination (Shonkoff et al., 2021). These adversities appear to have a synergistic effect on pubertal timing (Senger-Carpenter et al., 2023) and may uniquely affect the direction of pubertal timing based on the intersection of sociodemographic factors (Stenson et al., 2021). Further research is needed to disentangle these complex interrelationships.

## Conclusions

In summary, the current findings suggest that males and females may both be susceptible to negative mental health consequences and acceleration in biological aging following threatening early-life experiences. The findings also provide support for accelerated biological aging (measured through pubertal timing) as one pathway through which threat-related adversity may confer risk for psychopathology. While deprivation exposure was associated with mental health problems in both sexes, pubertal timing did not explain this association. The differing association with pubertal timing across threat and deprivation exposures provides support for dimensional models of adversity, which suggest that there may be distinct consequences of adversity exposure depending on the underlying characteristics of that adversity. Finally, the current finding that adolescents with greater exposure to ELA experience more mental health problems suggests that potential negative impacts of adversity may be present in early adolescence. Pubertal timing in both males and females may act as a risk indicator, necessitating further psychological assessment and support in early-maturing individuals.

## Supporting information

Shaul et al. supplementary material Shaul et al. supplementary material

## Supplementary material

The supplementary material for this article can be found at https://doi.org/10.1017/S003329172400179X
