# Supplementary material for: Pubertal timing mediates the association between threat adversity and psychopathology
Source: Psychol Med. 2024 Sep 26;54(12):3436–46. doi: 10.1017/S003329172400179X (PMC11496226; doi:10.1017/S003329172400179X)
Supplement: Shaul et al. supplementary material [file S003329172400179Xsup001.docx]

**Figure S1**

*Flow chart of Participant Exclusion and Analyses*

Threat, deprivation, SES, and household instability factor scores extracted for sub-sample with complete case data for all items included in the four-factor CFA

(*n* = 8003)

Participants with baseline data on any adversity variables of interest in release 4.0

(*N* = 11878)

Excluded due to missingness on any item included in the final threat or deprivation factors in CFA

(*n* = 913) and as recorded sex changed between timepoints

(*n* = 1)

Threat and deprivation latent factor scores extracted in the two-factor CFA

(complete case analyses; *n* = 10964)

Excluded due to missingness of site ID (*n* = 208) and all CBCL items for all timepoints in release 5.0 (*n* = 1069)

Imputation of PDS average and CBCL internalising and externalising problem scores

Primary mediation and conditional process analysis (threat and deprivation)

(*n* = 9687)

Imputation of four-factor adversity latent scores for supplementary analysis

Supplementary mediation analysis

*Note.* CFA, Confirmatory factor analysis; SES, Socioeconomic stressors; CBCL, Child Behavior Checklist; PDS, Pubertal Development Scale. Supplementary analysis indicated with dotted line.

**Table S1**

*Comparing Included and Excluded Participants on Key Baseline Variables*

|  | Included | Excluded | Comparison |
| --- | --- | --- | --- |
| *n* | 9687 | 2190 |  |
| Sex |  |  | $\chi^{2}$(2) = 2.11 |
| Female | 4602 (48%) | 1078 (49%) |  |
| Male | 5085 (52%) | 1112 (51%) |  |
| Race/Ethnicity |  |  | $\chi^{2}$(5) = 263.95** |
| White | 5333 (55%) | 850 (39%) | ** |
| Black | 1263 (13%) | 521 (24%) | ** |
| Hispanic | 1867 (19%) | 544 (25%) | ** |
| Asian | 205 (2%) | 47 (2%) |  |
| Other | 1019 (11%) | 228 (10%) |  |
| Age | 9.92 (.63) [8.92-11.08] | 9.92 (.63) [8.92-11] | *F*(1,11852) = 0.01 |
| Parent Education | 17 (2.6) [3-21] | 15 (3) [3-21] | *F*(1,11852) = 353.56** |
| PDS | 1.4 (0.55) [0-4] | 1.4 (0.57) [0-4] | *F*(1,11852) = 5.30* |
| Externalizing Problems | 4.3 (5.6) [0-49] | 5.3 (6.8) [0-47] | *F*(1,11852) = 60.70** |
| Internalizing Problems | 4.9 (5.4) [0-51] | 5.5 (6) [0-41] | *F*(1,11852) = 16.33** |
| Threat Exposure | 0.09 (0.53) [-0.46-3.1] | 0.15 (0.57) [-0.46-3.1] | *F*(1,10964) = 14.40** |
| Deprivation Exposure | 0.01 (0.63) [-2.4-1.1] | 0.06 (0.64) [-2.5-1.1] | *F*(1,10964) = 7.28* |

*Note.* Values in the *Included* and *Excluded* columns are presented in the following format: frequency/mean (proportion/standard deviation) [minimum, maximum]. Values in the *Comparison* column are presented in the following format: statistic (degrees of freedom). **p* < .05, ***p* < .001.

**Table S2**

*Questionnaires used from the Adolescent Behaviour and Cognitive Development Study in Confirmatory Factor Analysis*

| Questionnaire | Table Name | Details | Informant | Measure |
| --- | --- | --- | --- | --- |
| ABCD Longitudinal Tracking | abcd_lt01 | Site identifier | = | Unique identifier |
| Kiddie Schedule for Affective Disorders and Schizophrenia for DSM-5 (PTSD-Checklist) | abcd_ptsd01 | Lifetime occurance of traumatic events | Parent | 0 – “No”, 1 – “Yes“ |
| Kiddie Schedule for Affective Disorders and Schizophrenia for DSM-5 (Background) | dibf01 | School, family and social relations at baseline | Parent | 0 – “No”, 1 – “Yes“ |
| Family Environment Scale – Conflict Subscale | abcd_fes01 | Percieved degree of conflict expressed by most members of the family at baseline. Some items are reverse coded and have been adjusted so that higher scores relect greater conflict. | Youth | “True”, “False” |
| Children’s Reports of Parental Behavior Inventory | crpbi01 | Percieved acceptance from primary and secondary caregivers at baseline | Youth | 1 – “Not like them”, 3 – “A lot like them” |
| Parental Monitoring Questionnaire | pmq01 | Percieved degree of parental supervision at baseline | Youth | 1 – “Never’, 5 – “Always” |
| Demographics Survey | pdem02 | Experiences of material hardship within the past 12 months; income bracket of caregivers; education level of caregivers | Parent | 0 – “No”, 1 – “Yes”; 1 – “Less than $5000”, 10 – “$200,000 or greater”; 0 – “Never attended”, 21 – “Doctoral degree” |
| Family History Assessment | abcd_fhxssp01 | Summary scores from family history inventory assessing lifetime occurance of psychological problems in the child’s bioloigical relatives | Parent | 0 – “No problem endorsed”, 1 – “Problem endorsed" |
| Residential History Derived Scores | abcd_rhds01 | External environmental and neighborhood data linked to residential geocodes | *Automated* | *Item specific* |
| Neighborhood Safety and Crime | abcd_pnsc01 | Percieved level of neighborhood safety and crime at baseline | Parent | 1 – “Strongly disagree”, 5 – “Strongly agree" |

*Note.* All questionnaires taken from Realease 4.0. Parent self-report of the attained education level of themselves and a secondary caregiver were measured as an ordinal variable. Lower values represent lower attained education*.*

**Confirmatory Factor Analysis**

***Methods***

INR was calculated by first recoding the household income variable to reflect the mean dollar value of its corresponding income bracket. This mean income level was then divided by the 2018 U.S. Federal Poverty Guidelines (Department of Health and Human Services, January 18, 2018) based on the number of household members. Parent education was calculated as the mean education level the parent completing the survey and their partner. Parental separation was calculated as a binary variable where “0” indicated the child was living with their biological parents’ full time and “1” indicated that the child was living with non-biological parents at least part of the time and/or is adopted or living in custodial care. INR, ADI, and parent education were mean centred and scaled; all other items were treated as categorical and entered as raw scores into the CFA.

Prior to entry into CFA models, continuous variables were scaled. Data was randomly split into training (*n* = 5482) and testing (*n* = 5482) using the ‘set.seed’ function in the base package of *R (R Core Team, 2021)*. Analyses were conducted using the *lavaan* package (Rosseel, 2012) (version 0.6.13) in *RStudio (Team, 2020)* and estimated with weighted least-square with mean and variance correction (WLSMV)*.* Ordinal variables were specified, which in the *lavaan* package allows model parameter calculation using diagonally weighted least squares (DWLS), mean and variance adjusted test statistic, and computation of robust standard errors using the full weight matrix. Correlations between the factors were allowed, consistent with literature finding high co-occurrence of ELAs (Kim, Mennen, & Trickett, 2017).

As noted in the primary paper, the CFA was conducted in multiple stages. This began with an initial ‘pre-CFA’ stage that included separate modelling for four adversity factors (threat, deprivation, household instability, and SES), which was conducted with the training dataset (*n* = 5482). This ‘pre-CFA’ stage used a more lenient model fit criterion of CFI ≥ .90. Using the pre-CFA models, a combined four-factor model was submitted for CFA using the the subset of data, which was then submitted to the hold-out testing set (*n* = 5482) in the third stage. Then, the four-factor model was submitted to CFA using the full sample of participants with complete data for all items. Latent factor scores were extracted for this subset of participants and only used for the supplemental analyses (see Table S10). To make use of the larger dataset that had available data on threat and deprivation measures, the two-factor model of threat and deprivation (using the same items established in the four-factor model) was submitted to CFA on the larger sample of *n* = 10,964 and latent factor scores extracted for all participants.

***Results***

As reflected in Table S3, items from the demographic survey on family experiences of material deprivation were initially theorised as part of the deprivation factor. Due to low factor loading, these items were then tested in the SES factor where appropriate model fit was found in the ‘pre-CFA’ stage. Ten residual correlations were added at this stage between secondary caregiver items on the Children’s Reports of Parental Behavior Inventory based on modification indices. These were justified as each item originated from the same questionnaire and regarded the same caregiver. Model fit indices for individual factors from the ‘pre-CFA’ stage are presented in Table S4. When the combined four-factor model was submitted on the training dataset, items identified for issues with collinearity and items with low factor loadings were removed where it was considered that there was construct overlap with other items. This four-factor model was submitted for CFA using the hold-out testing set, which had appropriate model fit (Table S5). Subsequently, when the four-factor model was submitted for CFA on the entire sample, the covariance matrix of latent variables was found to be not positive definite due to the threat item *ksads_ptsd_raw_761_p* (“Has your child been shot, stabbed, or beaten brutally by a non-family member?”) that had a negative variance estimate and “NA” *R^2^* value. Inspection of the frequency distribution of this item reflected a likely floor effect, with 40 participants out of the total sample (0.004%) responding “Yes” to this question. After this item was removed, the model converged with no warnings and met appropriate model fit criteria (Table S5). Factor loading scores for individual items included in the final factors are presented in Table S6. Latent factor scores for the four-factors were extracted for each participant with complete case data (*n* = 8003). Finally, the two-factor model was submitted for CFA on the full dataset (see Table S5 for model fit), and latent factors score for threat and deprivation were extracted for participants with complete case data (*n* = 10964). Pearson’s correlation analysis found latent factor scores extracted from the two-factor and four-factor models were highly correlated (*r* > .90, *p* < .001) for corresponding adversity factors (Figure S2).

**Table S3**

*Proposed Formative Adversity Factors: ABCD Study Questionnaire Items and Modifications Made During Confirmatory Factor Analysis*

| Hypothesised Factor | Adversity Domain | Item | ABCD Item Name  (Survey short name) | Stage of Modifications | Reason |
| --- | --- | --- | --- | --- | --- |
| Threat | Physical Abuse | Beaten to the point of having bruises by a family member | ksads_ptsd_raw_763_p (abcd_ptsd01) |  |  |
|  |  | A non-family member threatened to kill your child | ksads_ptsd_raw_764_p (abcd_ptsd01) |  |  |
|  |  | A family member threatened to kill your child | ksads_ptsd_raw_765_p (abcd_ptsd01) |  |  |
|  |  | Shot, stabbed, or beaten brutally by a grown up in the home | ksads_ptsd_raw_762_p (abcd_ptsd01) | Training: Removed | > 1.00 implied correlation with KSADS items 763 & 761 |
|  |  | Shot, stabbed, or beaten brutally by a non-family member | ksads_ptsd_raw_761_p (abcd_ptsd01) | Full dataset: Removed | Negative residual variance |
|  | Sexual Abuse | A grown up in the home touched your child in their privates, had your child touch their privates, or did other sexual things to your child | ksads_ptsd_raw_767_p (abcd_ptsd01) |  |  |
|  |  | An adult outside your family touched your child in their privates, had your child touch their privates or did other sexual things to your child | ksads_ptsd_raw_768_p (abcd_ptsd01) |  |  |
|  |  | A peer forced your child to do something sexually | ksads_ptsd_raw_769_p (abcd_ptsd01) |  |  |
|  | Witnessing Violence | Witness the grownups in the home push, shove or hit one another | ksads_ptsd_raw_766_p (abcd_ptsd01) |  |  |
|  |  | Witnessed or present during an act of terrorism | ksads_ptsd_raw_758_p (abcd_ptsd01) |  |  |
|  |  | Witnessed death or mass destruction in a war zone | ksads_ptsd_raw_759_p (abcd_ptsd01) |  |  |
|  |  | Witnessed someone shot or stabbed in the community | ksads_ptsd_raw_760_p (abcd_ptsd01) |  |  |
|  |  | Family members sometimes get so angry they throw things | fes_youth_q3 (abcd_fes01) |  |  |
|  |  | Family members sometimes hit each other | fes_youth_q6 (abcd_fes01) |  |  |
|  | Bullying | Does your child have any problems with bullying at school or in your neighborhood? | kbi_p_c_bully  (dibf01) |  |  |
|  | Accidental Injury or Death | A car accident in which your child or another person in the car was hurt bad enough to require medical attention? | ksads_ptsd_raw_754_p (abcd_ptsd01) |  |  |
|  |  | Another significant accident for which your child needed specialized and intensive medical treatment? | ksads_ptsd_raw_755_p (abcd_ptsd01) |  |  |
|  |  | Witnessed or caught in a fire that caused significant property damage or personal injury | ksads_ptsd_raw_756_p (abcd_ptsd01) |  |  |
|  |  | Witnessed or caught in a natural disaster that caused significant property damage or personal injury | ksads_ptsd_raw_757_p (abcd_ptsd01) |  |  |
|  |  | Learned about the sudden unexpected death of a loved one | ksads_ptsd_raw_770_p (abcd_ptsd01) |  |  |
| Deprivation | Emotional Neglect | Makes me feel better after talking over my worries with him/her | crpbi_parent1_y; crpbi_caregiver12_y (crpbi01) |  |  |
|  |  | Smiles at me very often | crpbi_parent2_y; crpbi_caregiver13_y (crpbi01) |  |  |
|  |  | Is able to make me feel better when I am upset | crpbi_parent3_y; crpbi_caregiver14_y (crpbi01) |  |  |
|  |  | Believes in showing his/her love for me | crpbi_parent4_y; crpbi_caregiver15_y (crpbi01) |  |  |
|  |  | Is easy to talk to | crpbi_parent5_y; crpbi_caregiver16_y (crpbi01) |  |  |
|  |  | We fight a lot in our family [r] | fes_youth_q1 (abcd_fes01) |  |  |
|  |  | Family members rarely become openly angry | fes_youth_q2 (abcd_fes01) |  |  |
|  |  | Family members hardly ever lose their tempers | fes_youth_q4 (abcd_fes01) |  |  |
|  |  | Family members often criticize each other | fes_youth_q5 (abcd_fes01) |  |  |
|  |  | If there is a disagreement in our family, we try hard to smooth things over and keep the peace [r] | fes_youth_q7 (abcd_fes01) |  |  |
|  |  | Family members often try to one-up or outdo each other [r] | fes_youth_q8 (abcd_fes01) |  |  |
|  |  | In our family, we believe you don't ever get anywhere by raising your voice | fes_youth_q9 (abcd_fes01) |  |  |
|  | Physical Neglect | How often do your parents/guardians know where you are? | parent_monitor_q1_y (pmq01) |  |  |
|  |  | How often do your parents know who you are with when you are not at school and away from home? | parent_monitor_q2_y (pmq01) |  |  |
|  |  | If you are at home when your parents or guardians are not, how often do you know how to get in touch with them? | parent_monitor_q3_y (pmq01) |  |  |
|  |  | How often do you talk to your parent or guardian about your plans for the coming day, such as your plans about what will happen at school or what you are going to do with friends? | parent_monitor_q4_y (pmq01) |  |  |
|  |  | In an average week, how many times do you and your parents/guardians, eat dinner together? | parent_monitor_q5_y (pmq01) |  |  |
|  | Material Deprivation | Needed food but couldn't afford to buy it or couldn't afford to go out to get it? | demo_fam_exp1_v2_l (pdem02) | Pre-CFA: Moved to SES factor | < .30 factor loading |
|  |  | Were without telephone service because you could not afford it? | demo_fam_exp2_v2_l (pdem02) | Pre-CFA: Moved to SES factor | < .30 factor loading |
|  |  | Didn't pay the full amount of the rent or mortgage because you could not afford it? | demo_fam_exp3_v2_l (pdem02) | Pre-CFA: Moved to SES factor | < .30 factor loading |
|  |  | Were evicted from your home for not paying the rent or mortgage? | demo_fam_exp4_v2_l (pdem02) | Pre-CFA: Moved to SES factor | < .30 factor loading |
|  |  | Had services turned off by the gas or electric company, or the oil company wouldn't deliver oil because payments were not made? | demo_fam_exp5_v2_l (pdem02) | Pre-CFA: Moved to SES factor | < .30 factor loading |
|  |  | Had someone who needed to see a doctor or go to the hospital but didn't go because you could not afford it? | demo_fam_exp6_v2_l (pdem02) | Pre-CFA: Moved to SES factor | < .30 factor loading |
|  |  | Had someone who needed a dentist but couldn't go because you could not afford it? | demo_fam_exp7_v2_l (pdem02) | Pre-CFA: Moved to SES factor | < .30 factor loading |
| Household Instability | Parental Separation | Child lives with non-biological parents or other caregivers at least part of the time, is adopted or in custodial care | kbi_p_c_guard__[3-12] (dibf01); demo_prim  (pdem02) |  |  |
|  | Caregiver Substance Abuse | Overall parents alcohol problem | famhx_ss_parent_alc_p (abcd_fhxssp01) |  |  |
|  |  | Overall parents drug use problem | famhx_ss_parent_dg_p (abcd_fhxssp01) |  |  |
|  | Caregiver Mental Illness | Overall parents depression problem | famhx_ss_parent_dprs_p (abcd_fhxssp01) |  |  |
|  |  | Overall parents mania problem | famhx_ss_parent_ma_p (abcd_fhxssp01) |  |  |
|  |  | Overall parents visions of others spying/plotting problem | famhx_ss_parent_vs_p (abcd_fhxssp01) | Pre-CFA: Removed | < .30 factor loading |
|  |  | Overall parents nerves/nervous breakdown problem | famhx_ss_parent_nrv_p (abcd_fhxssp01) |  |  |
|  |  | Overall parents hospitalized due to emotional/mental problem | famhx_ss_parent_hspd_p (abcd_fhxssp01) |  |  |
|  |  | Overall parents attempted or committed suicide | famhx_ss_parent_scd_p (abcd_fhxssp01) |  |  |
|  |  | Overall parents trouble holds job/fights/police problem | famhx_ss_parent_trb_p (abcd_fhxssp01) |  |  |
| Socio-economic Stressors | Income-to-needs ratio (INR) | Total family income divided by poverty threshold based on household size | demo_comb_income_v2; demo_roster_v2 (pdem02) |  |  |
|  | Parent education | Mean score of parent and partner highest grade or level of school completed or the highest degree received | demo_prnt_ed_v2_l demo_prtnr_ed_v2_l (pdem02) |  |  |
|  | Neighbourhood disadvantage | Area Deprivation Index: national percentiles | reshist_addr1_adi_perc (abcd_rhds01) |  |  |
|  |  | I feel safe walking in my neighborhood, day or night (r) | neighborhood1r_p (abcd_pnsc01) | Training: Removed | Lowest factor loading |
|  |  | Violence is not a problem in my neighborhood (r) | neighborhood2r_p (abcd_pnsc01) | Training: Removed | Lowest factor loading |
|  |  | My neighborhood is safe from crime (r) | neighborhood3r_p (abcd_pnsc01) | Training: Removed | Lowest factor loading |
|  | Material Deprivation | Needed food but couldn't afford to buy it or couldn't afford to go out to get it? | demo_fam_exp1_v2_l (pdem02) | Training: Removed | > 1.00 implied correlation with INR |
|  |  | Were without telephone service because you could not afford it? | demo_fam_exp2_v2_l (pdem02) | Training: Removed | > 1.00 implied correlation with INR |
|  |  | Didn't pay the full amount of the rent or mortgage because you could not afford it? | demo_fam_exp3_v2_l (pdem02) | Training: Removed | > 1.00 implied correlation with INR |
|  |  | Were evicted from your home for not paying the rent or mortgage? | demo_fam_exp4_v2_l (pdem02) | Training: Removed | > 1.00 implied correlation with INR |
|  |  | Had services turned off by the gas or electric company, or the oil company wouldn't deliver oil because payments were not made? | demo_fam_exp5_v2_l (pdem02) | Training: Removed | > 1.00 implied correlation with INR |
|  |  | Had someone who needed to see a doctor or go to the hospital but didn't go because you could not afford it? | demo_fam_exp6_v2_l (pdem02) | Training: Removed | > 1.00 implied correlation with INR |
|  |  | Had someone who needed a dentist but couldn't go because you could not afford it? | demo_fam_exp7_v2_l (pdem02) | Training: Removed | > 1.00 implied correlation with INR |

*Note.* CFA, Confirmatory Factor Analysis; Pre-CFA, factors entered individually into a CFA on “training” subset of data; Training, Four factors entered to CFA using “training” subset of data; Full dataset, Four factors entered to CFA using entire dataset.

**Table S4**

*Goodness-of-Fit Indicators of Individual Factor Adversity Models*

|  | Threat | Deprivation | SES | Instability |
| --- | --- | --- | --- | --- |
| Model Fit | χ^2^(153, *n* = 5482) = 1518.679*, RMSEA [90% CI] = .013 [.010, .015], CFI = .912 | χ^2^(199, *n* = 5482) = 2351.505*, RMSEA [90% CI] = .044 [.043, .046], CFI = .906 | χ^2^(58, *n* = 4591) = 536.013*, RMSEA [90% CI] = .042 [.039, .046], CFI = .994 | χ^2^(35, *n* = 4706) = 190.214*, RMSEA [90% CI] = .031 [.027, .035], CFI = .942 |

*Note.* SES, Socioeconomic stressors; RMSEA, root mean square error of approximation; CFI, comparative fit index; **p* < .001.

**Table S5**

*Goodness-of-Fit Indicators of Two and Four-Factor Adversity Models*

| Model | Training | Testing | Full Data |
| --- | --- | --- | --- |
| Two Factor | - | - | χ^2^(654, *n* = 10964) = 10520.870*, RMSEA [90% CI] = .037 [.036, .038], CFI = .978 |
| Four Factor | χ^2^(1159, *n* = 4014) = 6587.46*, RMSEA [90% CI] = .034 [.033, .035], CFI = .966 | χ^2^(1159, *n* = 3989) = 6689.08*, RMSEA [90% CI] = .035 [.034, .035], CFI = .966 | χ^2^(1158, *n* = 8003) = 12136.181*, RMSEA [90% CI] = .034 [.034, .035], CFI = .965 |

*Note.* RMSEA, root mean square error of approximation; CFI, comparative fit index; **p* < .001. All factors were significantly correlated at the *p* < .001 level. Threat-Deprivation (two factor: *r* = .15; four factor: *r* = .15); Threat-SES (*r* = .35); Threat-Instability (*r* = .47); Deprivation-SES (*r* = .21); Deprivation-Instability (*r* = .17); SES-Instability (*r* = .40).

**Table S6**

*Factor loadings for adversity items in final four-factor and two-factor models*

| Factor | Item Description | Two-Factor | | | Four-Factor | | |
| --- | --- | --- | --- | --- | --- | --- | --- |
|  |  | Unstd | SE | Std | Unstd | SE | Std |
| Threat | Beaten to the point of having bruises by a family member | 1.00 | - | .86 | 1.00 | - | .88 |
|  | Death threat by non-family member | 1.01 | .02 | .87 | 0.96 | .03 | .85 |
|  | Death threat by family member | 1.06 | .03 | .91 | 1.03 | .03 | .90 |
|  | Sexual abuse by a family member | 1.11 | .03 | .96 | 1.10 | .03 | .97 |
|  | Sexual abuse by a non-family member | 1.04 | .02 | .89 | 1.02 | .03 | .89 |
|  | Sexual abuse by a peer | 0.89 | .02 | .76 | 0.84 | .03 | .74 |
|  | Witnessed domestic violence | 0.73 | .03 | .63 | 0.94 | .04 | .82 |
|  | Witnessed act of terrorism | 1.14 | .03 | .98 | 1.09 | .03 | .95 |
|  | Witnessed death or destruction in war zone | 1.09 | .03 | .94 | 1.06 | .03 | .93 |
|  | Witnessed shooting or stabbing in community | 0.96 | .02 | .82 | 0.95 | .02 | .83 |
|  | Family members throwing objects in anger | 0.41 | .04 | .36 | 0.43 | .04 | .37 |
|  | Experienced bullying | 0.36 | .04 | .31 | 0.49 | .03 | .43 |
|  | Experienced serious car accident | 0.57 | .03 | .49 | 0.45 | .04 | .39 |
|  | Witnessed/experienced fire causing damage/injury | 0.72 | .03 | .62 | 0.63 | .03 | .56 |
|  | Witnessed/experienced natural disaster causing damage/injury | 0.70 | .03 | .61 | 0.61 | .03 | .54 |
|  | Learned of sudden/unexpected death of loved one | 0.42 | .03 | .36 | 0.46 | .03 | .41 |
| Deprivation | Makes me feel better after talking over my worries with him/her (primary) | 1.00 | - | .71 | 1.00 | - | .68 |
|  | Makes me feel better after talking over my worries with him/her (secondary) | 0.53 | .02 | .38 | 0.58 | .02 | .39 |
|  | Smiles at me very often (primary parent) | 0.91 | .02 | .64 | 0.94 | .02 | .64 |
|  | Smiles at me very often (other caregiver) | 0.54 | .02 | .39 | 0.62 | .02 | .42 |
|  | Is able to make me feel better when I am upset (primary parent) | 1.05 | .02 | .74 | 1.05 | .02 | .72 |
|  | Is able to make me feel better when I am upset (other caregiver) | 0.58 | .02 | .41 | 0.63 | .02 | .43 |
|  | Believes in showing his/her love for me (primary parent) | 1.07 | .02 | .76 | 1.11 | .03 | .75 |
|  | Believes in showing his/her love for me (other caregiver) | 0.54 | .02 | .38 | 0.62 | .03 | .42 |
|  | Is easy to talk to (primary parent) | 0.91 | .02 | .64 | 0.93 | .02 | .63 |
|  | Is easy to talk to (other caregiver) | 0.50 | .02 | .35 | 0.54 | .02 | .37 |
|  | We fight a lot in our family [r] | -0.78 | .02 | -.55 | -0.84 | .03 | -.57 |
|  | Family members rarely become openly angry | -0.57 | .02 | -.4 | -0.61 | .03 | -.42 |
|  | Family members hardly ever lose their tempers | -0.63 | .02 | -.45 | -0.67 | .03 | -.46 |
|  | Family members often criticize each other | -0.69 | .02 | -.49 | -0.73 | .03 | -.50 |
|  | If there is a disagreement in our family, we try hard to smooth things over and keep the peace [r] | -0.71 | .03 | -.50 | -0.74 | .03 | -.51 |
|  | Family members often try to one-up or outdo each other [r] | -0.52 | .02 | -.37 | -0.54 | .03 | -.37 |
|  | In our family, we believe you don't ever get anywhere by raising your voice | -0.47 | .02 | -.33 | -0.49 | .03 | -.33 |
|  | How often do your parents/guardians know where you are? | 0.62 | .02 | .44 | 0.65 | .03 | .44 |
|  | How often do your parents know who you are with when you are not at school and away from home? | 0.55 | .02 | .39 | 0.58 | .03 | .40 |
|  | If you are at home when your parents or guardians are not, how often do you know how to get in touch with them? | 0.47 | .02 | .33 | 0.48 | .02 | .32 |
|  | How often do you talk to your parent or guardian about your plans for the coming day, such as your plans about what will happen at school or what you are going to do with friends? | 0.52 | .02 | .37 | 0.52 | .02 | .35 |
|  | In an average week, how many times do you and your parents/guardians, eat dinner together? | 0.46 | .02 | .33 | 0.49 | .02 | .34 |
| SES | Income to needs ratio | - | - | - | 1.00 | - | .90 |
|  | Area deprivation index | - | - | - | -18.28 | .58 | -16.35 |
|  | Parent education | - | - | - | 0.66 | .02 | .60 |
| Household Instability | Overall parents alcohol problem | - | - | - | 1.00 | - | .63 |
|  | Overall parents drug use problem | - | - | - | 0.96 | .03 | .61 |
|  | Overall parents depression problem | - | - | - | 1.30 | .03 | .83 |
|  | Overall parents mania problem | - | - | - | 1.17 | .04 | .75 |
|  | Overall parents nerves/nervous breakdown problem | - | - | - | 0.93 | .04 | .59 |
|  | Overall parents hospitalized due to emotional/mental problem | - | - | - | 1.28 | .04 | .82 |
|  | Overall parents attempted or committed suicide | - | - | - | 1.21 | .04 | .77 |
|  | Overall parents trouble holds job/fights/police problem | - | - | - | 1.35 | .03 | .86 |
|  | Separation from parents due to adoption, removal child protection or divorce | - | - | - | 0.72 | .05 | .46 |

*Note.* Unstd, Unstandardised estimate; SE, Standard error; Std, Standardised estimate*;* SES, Socioeconomic stressors; r, reverse scored item.

**Figure S2**

*Associations of Adversity Latent Scores from Two and Four Factor Models Pre and Post Imputation*

*Note.* The matrix presents Person’s *r* correlations between latent scores of two- and four-factor confirmatory factor analyses. Corresponding factor analyses are represented by suffixes “_2” and “_4” for scores from the two- and four-factor analyses respectively. Suffixes “_pre” and “_post” respectively reflect four-factor latent scores prior to and after imputation. All associations were significant at the *p* < .001 level.

**Imputation**

Baseline body mass was included as a predictor in the imputation model. This was calculated as the weight in kilograms devided by height in meters squared using baseline information from Youth Anthropometrics datafile (abcd_ant01.txt). Imputation was conducted using the *mice* package (Buuren & Groothuis-Oudshoorn, 2011).

***Code***

## SET UP

packages <- c("tidyverse","data.table","corrplot","caret","mice","doParallel","doRNG", "VIM", "mitml", "gtsummary")

if (length(setdiff(packages, rownames(installed.packages()))) > 0) {

install.packages(setdiff(packages, rownames(installed.packages()))) }

lapply(packages, library, character.only = TRUE)

nCores <- min(parallel::detectCores(), 8)

options(mc.cores = nCores)

options(cores = nCores)

doParallel::registerDoParallel(cores = nCores)

cat("### Using", foreach::getDoParWorkers(), "cores\n")

cat("### Using", foreach::getDoParName(), "as backend\n")

### Run mice with 0 iterations

imp <- mice(impute_data, maxit = 0)

### Extract predictorMatrix and methods of imputation

meth <- imp$method

predM <- imp$predictorMatrix

### Specify variables not to be predictors

predM[,c("subjectkey")] = 0

predM[,c("site_id_l")] = 0

predM[,c("rel_family_id")] = 0

predM[,c("race_ethnicity")] = 0

### Specify variables not to be imputed

meth[c("race_ethnicity")]= ""

meth[c("subjectkey")] = ""

meth[c("site_id_l")] = ""

meth[c("rel_family_id")] = ""

meth[c("threat_2")] = ""

meth[c("deprivation_2")] = ""

meth[c("sex")] = ""

## IMPUTATION

sample="randomsingle"

set.seed=999

### Set number of imputations (num) to match percentage of missing data

num = (sum(is.na(impute_data))/prod(dim(impute_data)))*100

### Run imputation

imputed <- mice(impute_data, maxit = 10, predictorMatrix = predM, method = meth, m = num , print = F)

## CREATE MEAN OF IMPUTED DATASETS

imputed_complete_long <- mice::complete(imputed, action = "long")

df <- imputed_complete_long %>% filter(.imp == 1) %>% select(.id, subjectkey, race_ethnicity, covid, site_id_l, rel_family_id, threat_2, deprivation_2, sex)

agg <- imputed_complete_long %>% select(-c(subjectkey, race_ethnicity, covid, site_id_l, rel_family_id, threat_2, deprivation_2, sex))

agg <- aggregate(agg, by = list(agg$.id), FUN = mean)

df_imp <- df %>% left_join(., agg, by = ".id") %>% select(-c(Group.1, .imp, .id))

**Table S7**

*Comparison of Observed and Imputed Variables*

|  |  | Observed |  |  |  | Imputed |  |
| --- | --- | --- | --- | --- | --- | --- | --- |
|  | *n* | M (SD) | Min-Max | % NA |  | M (SD) | Min-Max |
| *Female* | 4602 |  |  |  |  |  |  |
| PDS [2-year] | | 2.8 (0.6) | 1-4 | 0.02 |  | 2.8 (0.6) | 1-4 |
| Internalizing [3-year] | | 5.6 (6.1) | 0-49 | 0.26 |  | 5.6 (5.8) | 0-49 |
| Externalizing [3-year] | | 3.4 (4.9) | 0-43 | 0.26 |  | 3.4 (4.7) | 0-43 |
| *Male* | 5085 |  |  |  |  |  |  |
| PDS [2-year] | | 2.2 (0.6) | 1-4 | 0.02 |  | 2.2 (0.6) | 1-4 |
| Internalizing [3-year] | | 4.7 (5.5) | 0-44 | 0.26 |  | 4.6 (5.2) | 0-44 |
| Externalizing [3-year] | | 4.3 (5.8) | 0-48 | 0.26 |  | 4.3 (5.6) | 0-48 |

*Note. n*, number of participants in sample; M, mean; SD, standard deviation; Min, minimum; Max, maximum; % NA, percentage of missing data; PDS, Pubertal Development Scale.

## Table S8

*Correlations of Pubertal Timing with Adversity and Mental Health Problems by Sex*

|  | Pubertal Timing | Internalizing 3 year | Internalizing Baseline | Externalizing 3 year | Externalizing Baseline | Threat | Deprivation |
| --- | --- | --- | --- | --- | --- | --- | --- |
| Males | | | | | | | |
| Pubertal Timing | - | 0.05** | 0.04* | 0.07** | 0.05** | 0.06** | 0.01 |
| Internalizing 3 year | 0.05** | - | 0.63** | 0.59** | 0.42** | 0.19** | 0.09** |
| Internalizing Baseline | 0.04* | 0.63** | - | 0.39** | 0.57** | 0.25** | 0.11** |
| Externalizing 3 year | 0.07** | 0.59** | 0.39** | - | 0.67** | 0.23** | 0.11** |
| Externalizing Baseline | 0.05** | 0.42** | 0.57** | 0.67** | - | 0.28** | 0.15** |
| Threat | 0.06** | 0.19** | 0.25** | 0.23** | 0.28** | - | 0.27** |
| Deprivation | 0.01 | 0.09** | 0.11** | 0.11** | 0.15** | 0.27** | - |
| Females | | | | | | | |
| Pubertal Timing | - | 0.08** | 0.03** | 0.06** | 0.04** | 0.06** | 0.05** |
| Internalizing 3 year | 0.08** | - | 0.59** | 0.56** | 0.37** | 0.18** | 0.07** |
| Internalizing Baseline | 0.03* | 0.59** | - | 0.38** | 0.59* | 0.24** | 0.09** |
| Externalizing 3 year | 0.06** | 0.56** | 0.38** | - | 0.64** | 0.23** | 0.15** |
| Externalizing Baseline | 0.04* | 0.37** | 0.59** | 0.64** | - | 0.29** | 0.18** |
| Threat | 0.06** | 0.18** | 0.24** | 0.23** | 0.29** | - | 0.29** |
| Deprivation | 0.05** | 0.07** | 0.09** | 0.15** | 0.18** | 0.29** | - |

*Note.* Correlations between variables estimated by Pearson’s *r* values. **p* < .01; ***p* < .001.

**Figure S3**

**Mediation & Moderated Mediation**

Mediation and moderated mediation conducted using *brms* package using the syntax for multivariate multilevel models (Bürkner, 2017). Weakly informative conjugate priors were used for all parameters, allowing the observed data to have greater influence on the posterior distribution on which inference was based (Gelman, Simpson, & Betancourt, 2017; Yuan & MacKinnon, 2009). Weakly informative Student’s *t* priors (𝜇 = 0, 𝜎 = 1, *𝜈 =* 3) were specified for the population-level (fixed effect) parameters and *brms* default priors were deemed appropriate for remaining parameters (Vehtari, 2024). Posterior distributions were estimated with Markov chain Monte Carlo simulations. Samples were derived by 4 Markov chains with 15000 iterations (2000 warm-up samples). Where indicated to facilitate chain convergence, target proposal acceptance and number of trees evaluated were increased to .97 and 15 respectively. Adequate model convergence was assessed by Gelman-Rubin statistic (Rhat) values < 1.01, Effective Sample Sizes (ESS) values > 400 and visual inspection of the posterior predictive checks (Vehtari, Gelman, Simpson, Carpenter, & Bürkner, 2021).

***Code for Mediation Models***

#Model a1 predicts the *a* path wherein pubertal timing is predicted by adversity exposure (and covariates). A Gaussian response distribution is selected for pubertal timing (using the ‘family’ term), given the normal distribution of this variable (see Figure S3)

model_a1 = bf(pubertal_timing ~ 1 + adversity + sex + race_ethnicity + covid + (1|ID1|site_id_l) + (1|ID2|rel_family_id), family = “gaussian”)

#Model b1 predicts both the *b* and *c’* paths. Mental health problems are predicted by pubteral timing (*b* path) and adversity (*c’* path) (and covariates). A Poisson response distribution is selected for mental health problems (using the ‘family’ term), given the non-normal distribution of the CBCL data (see Figure S3)

model_b1 = bf(mental_health_problems ~ 1 + adversity + pubertal_timing + sex + covid + race_ethnicity + (1|ID1|site_id_l) + (1|ID2|rel_family_id), family = “poisson”)

#Models a1 and b1 are simultaneously estimated and population-level effects prior specified

model_mediation <- brm(data = brms_df, model_a1 + model_b1 + set_rescor(F), cores = 4, warmup=2000, iter=15000, thin=5, prior = prior(student_t(3, 0, 1), class = “b”), control = list(adapt_delta = 0.9))

#Mean and 95% quantile intervals of the regression coefficients extracted from the posterior distributions

post_model <- posterior_samples(model_mediation) %>%

mutate(a1 = b_pubertaltiming_adversity) %>%

mutate(b1 = b_mentalhealthproblems_pubertal_timing) %>%

mutate(indirect = a1*b1) %>%

mutate(direct = b_mentalhealthproblems_adversity) %>%

mutate(total = direct + indirect) %>%

mutate(proportion_mediated = indirect/total)

model_mediation_output <- post_model %>%

pivot_longer(c(a1, b1, direct, indirect, total)) %>%

group_by(name) %>%

mean_qi(value, .width = .95) %>%

mutate_if(is.double, round, digits = 3)

***Code for Moderated Mediation Models***

#Note: Using *brms,* all main effects are inherently calculated for variables included in interaction terms and do not need to be specified separately

#Model a1 (above) is adjusted to include interaction term of the predictor (adversity) and proposed moderator (sex)

model_moderation_a3 <- bf(pubertal_timing ~ 1 + adversity*sex + covid + race_ethnicity + (1|ID1|site_id_l) + (1|ID2|rel_family_id), family = “gaussian”)

#Model b1 (above) is adjusted to include interaction terms for the proposed moderator (sex) with the proposed mediator (pubertal timing) as well as the predictor (adversity)

model_moderation_b3 <- bf(mental_health_problems ~ pubertal_timing*sex + adversity*sex + covid + race_ethnicity + (1|ID1|site_id_l) + (1|ID2|rel_family_id), family = “poisson”)

#Models a3 and b3 are simultaneously estimated

model_moderation <- brm(data = brms_df, model_moderation_a3 + model_moderation _b3 + set_rescor(F), cores = 4, warmup=2000, iter=10000, thin=5, prior = prior(student_t(3, 0, 1), class = “b”), control = list(adapt_delta = 0.9))

#Mean and 95% quantile intervals of the regression coefficients extracted from the posterior distributions. Conditional effect equations from (Hayes & Rockwood, 2019)

post_model_moderation <- posterior_samples(model_moderation) %>%

mutate(

`direct_a when W is F` = (b_pubertaltiming_adversity+`b_pubertaltiming_deprivation_2:sexM` * 1),

`direct_a when W is M` = (b_pubertaltiming_adversity+ `b_pubertaltiming_deprivation_2:sexM` * 2),

`direct_b when W is F` = (b_mentalhealthproblems_pubertaltiming+

`b_mentalhealthproblems_pds_average_resid:sexM` * 1),

`direct_b when W is M` = (b_mentalhealthproblems_pubertaltiming+

`b_mentalhealthproblems_pds_average_resid:sexM` * 2),

`direct_c when W is F` = (b_mentalhealthproblems_adversity+

`b_mentalhealthproblems_sexM:deprivation_2` * 1),

`direct_c when W is M` = (b_mentalhealthproblems_adversity+

`b_mentalhealthproblems_sexM:deprivation_2` * 2),

`direct_difference_a` = `direct_a when W is F` - `direct_a when W is M`,

`direct_difference_b` = `direct_b when W is F` - `direct_b when W is M`,

`direct_difference_c` = `direct_c when W is F` - `direct_c when W is M`,

`first_stage when W is F` = (b_pubertaltiming_adversity+ `b_pubertaltiming_deprivation_2:sexM` * 1)

* b_mentalhealthproblems_pds_average_resid,

`first_stage when W is M` = (b_pubertaltiming_adversity+ `b_pubertaltiming_deprivation_2:sexM` * 2)

* b_mentalhealthproblems_pds_average_resid,

`first_stage_difference` = `first_stage when W is F` - `first_stage when W is M`,

`second_stage when W is F` = b_pubertaltiming_adversity*

(b_mentalhealthproblems_pubertaltiming+

`b_mentalhealthproblems_pds_average_resid:seM` * 1),

`second_stage when W is M` = b_pubertaltiming_adversity*

(b_mentalhealthproblems_pubertaltiming+ `b_mentalhealthproblems_pds_average_resid:sexM` * 2),

`second_stage_difference` = `second_stage when W is F` - `second_stage when W is M`,

`first_second_stage_F`=

(b_pubertaltiming_deprivation_2*b_mentalhealthproblems_pds_average_resid) + ((b_pubertaltiming_deprivation_2*`b_mentalhealthproblems_pds_average_resid:sexM`)+

(`b_pubertaltiming_deprivation_2:sexM`*b_mentalhealthproblems_pds_average_resid)*1) + ((`b_pubertaltiming_deprivation_2:sexM`*`b_mentalhealthproblems_pds_average_resid:sexM`)*1^2),

`first_second_stage_M`=

(b_pubertaltiming_deprivation_2*b_mentalhealthproblems_pds_average_resid) +

((b_pubertaltiming_deprivation_2*`b_mentalhealthproblems_pds_average_resid:sexM`)+

(`b_pubertaltiming_deprivation_2:sexM`*b_mentalhealthproblems_pds_average_resid)*2) + ((`b_pubertaltiming_deprivation_2:sexM`*`b_mentalhealthproblems_pds_average_resid:sexM`)*2^2),

`firstsecondstage_difference` = `first_second_stage_F` - `first_second_stage_M`)

output _mod <- post_model_moderation %>%

pivot_longer(contains("difference")) %>%

group_by(name) %>%

mean_qi(value, .width = c(.95, .99)) %>%

mutate_if(is.double, round, digits

**Figure S4**

*Posterior Predictive Checks for Mediation Models*

**
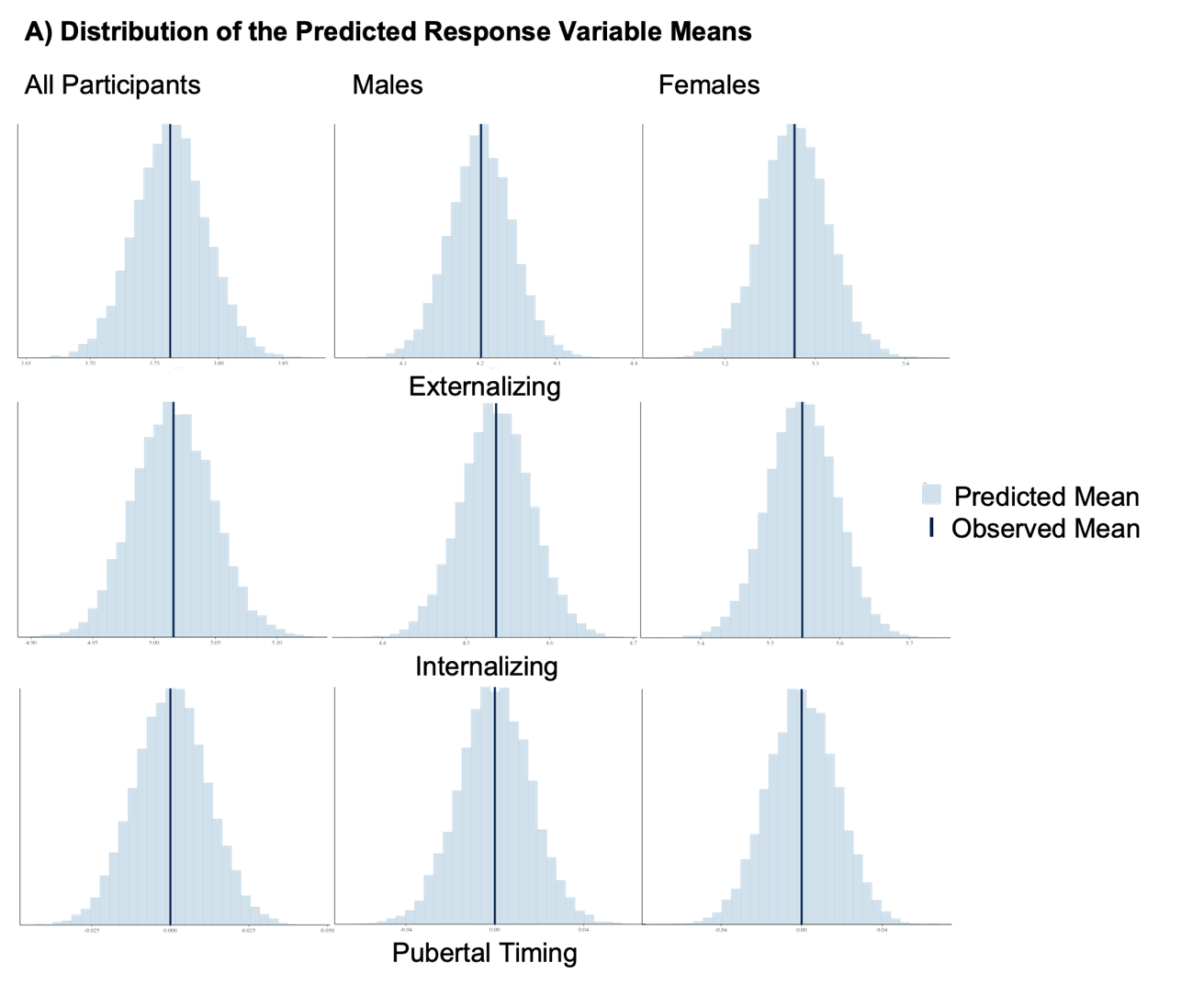

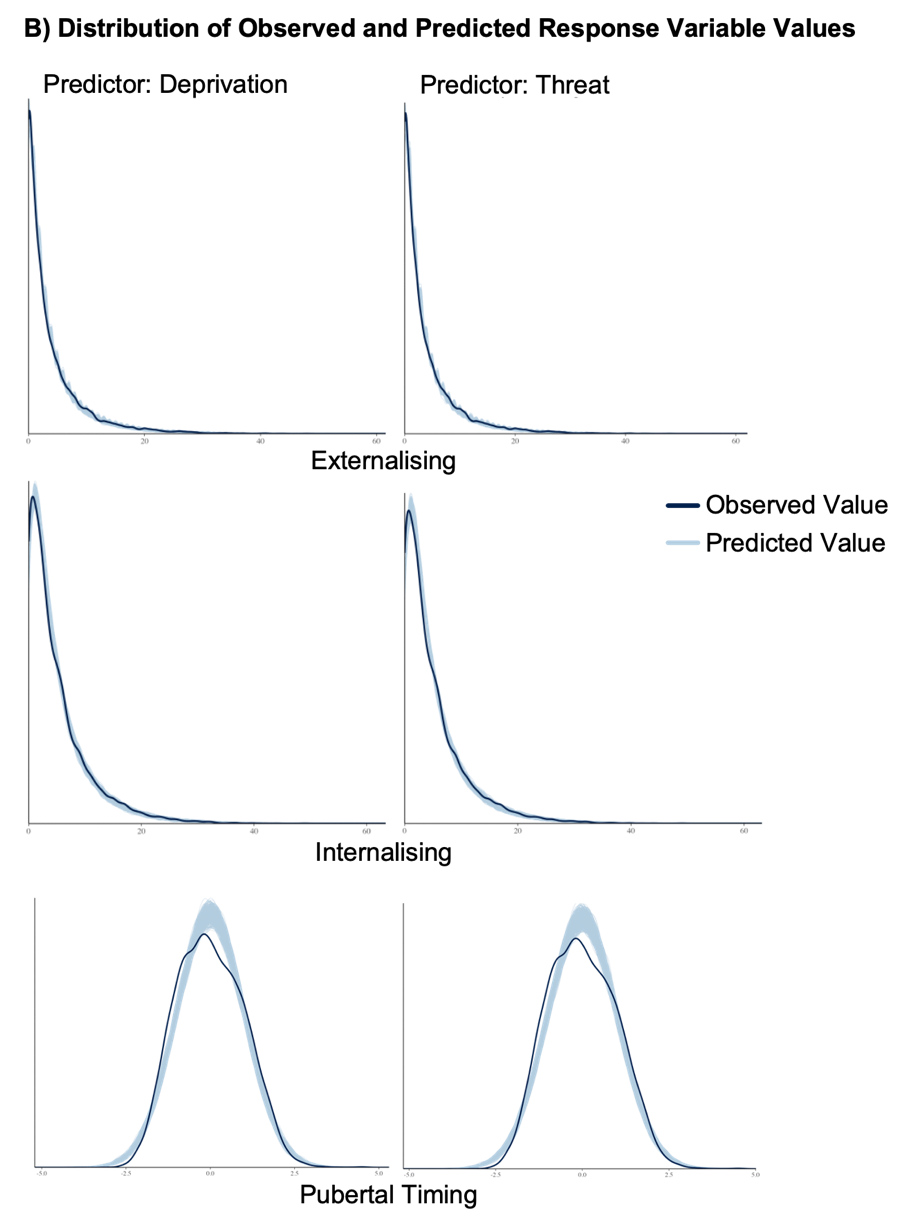
**

**Table S9**

*Mediating Effects of Pubertal Timing on Adversity Exposure and Mental Health Problems from Primary and Sensitivity Analyses*

|  | | Adversity – Pubertal Timing | | |  | Pubertal Timing – MH Problems | | |  | Adversity – MH Problems | | |  | Indirect Effect of Pubertal Timing | | |  | Proportion Mediated | | |
| --- | --- | --- | --- | --- | --- | --- | --- | --- | --- | --- | --- | --- | --- | --- | --- | --- | --- | --- | --- | --- |
|  | Est | | 99% CI L | 99% CI U |  | Est | 99% CI L | 99% CI U |  | Est | 99% CI L | 99% CI U |  | Est | 99% CI L | 99% CI U |  | Est | 99% CI L | 99% CI U |
| Threat |  | |  |  |  |  |  |  |  |  |  |  |  |  |  |  |  |  |  |  |
| Internalizing |  | |  |  |  |  |  |  |  |  |  |  |  |  |  |  |  |  |  |  |
| Primary | **0.09826** | | 0.03687 | 0.16028 |  | **0.03989** | 0.00137 | 0.07943 |  | **0.41443** | 0.35101 | 0.47491 |  | **0.00393** | 0.00014 | 0.00992 |  | 0.00942 | 0.00035 | 0.02319 |
| Baseline | **0.06955** | | 0.01771 | 0.12193 |  | **0.06761** | 0.02830 | 0.10654 |  | **0.08391** | 0.04132 | 0.12817 |  | **0.00471** | 0.00096 | 0.00996 |  | 0.05512 | 0.01083 | 0.14100 |
| Deprivation | **0.07984** | | 0.02923 | 0.13116 |  | **0.04166** | 0.00362 | 0.08013 |  | **0.28875** | 0.23739 | 0.34120 |  | **0.00334** | 0.00023 | 0.00824 |  | 0.01148 | 0.00075 | 0.02871 |
| Parent Ed | **0.09084** | | 0.02832 | 0.15344 |  | **0.04135** | 0.00306 | 0.07970 |  | **0.41676** | 0.35423 | 0.47829 |  | **0.00377** | 0.00022 | 0.00952 |  | 0.00899 | 0.00058 | 0.02293 |
| Externalizing |  | |  |  |  |  |  |  |  |  |  |  |  |  |  |  |  |  |  |  |
| Primary | **0.09442** | | 0.03418 | 0.15962 |  | 0.02141 | -0.02210 | 0.06421 |  | **0.53808** | 0.46240 | 0.61275 |  | 0.00204 | -0.00209 | 0.00757 |  | - | - | - |
| Baseline | **0.07193** | | 0.01876 | 0.12360 |  | **0.05044** | 0.00602 | 0.09414 |  | **0.13014** | 0.07827 | 0.18168 |  | **0.00363** | 0.00029 | 0.00861 |  | 0.02774 | 0.00221 | 0.07297 |
| Deprivation | **0.07665** | | 0.02457 | 0.12713 |  | 0.02168 | -0.02158 | 0.06396 |  | **0.34535** | 0.28373 | 0.40401 |  | 0.00167 | -0.00166 | 0.00591 |  | - | - | - |
| Parent Ed | **0.08623** | | 0.02331 | 0.14850 |  | 0.02063 | -0.02237 | 0.06297 |  | **0.53226** | 0.45654 | 0.60599 |  | 0.00181 | -0.00193 | 0.00707 |  | - | - | - |
| Deprivation |  | |  |  |  |  |  |  |  |  |  |  |  |  |  |  |  |  |  |  |
| Internalizing |  | |  |  |  |  |  |  |  |  |  |  |  |  |  |  |  |  |  |  |
| Primary | 0.02060 | | -0.02028 | 0.06193 |  | **0.04095** | 0.00320 | 0.07888 |  | **0.11593** | 0.07686 | 0.15516 |  | 0.00085 | -0.00088 | 0.00337 |  | - | - | - |
| Baseline* | 0.01640 | | -0.02470 | 0.05761 |  | **0.06801** | 0.02669 | 0.10739 |  | **0.03981** | 0.00466 | 0.07538 |  | 0.00112 | -0.00178 | 0.00461 |  | - | - | - |
| Threat | 0.00307 | | -0.03995 | 0.04657 |  | **0.04199** | 0.00359 | 0.07998 |  | **0.05553** | 0.01409 | 0.09651 |  | 0.00013 | -0.00202 | 0.00236 |  | - | - | - |
| Parent Ed | 0.01602 | | -0.02517 | 0.05830 |  | **0.04067** | 0.00172 | 0.07928 |  | **0.11515** | 0.07598 | 0.15475 |  | 0.00066 | -0.00116 | 0.00314 |  | - | - | - |
| Externalizing |  | |  |  |  |  |  |  |  |  |  |  |  |  |  |  |  |  |  |  |
| Primary | 0.01851 | | -0.02262 | 0.06093 |  | 0.02360 | -0.01814 | 0.06562 |  | **0.20939** | 0.16193 | 0.25696 |  | 0.00045 | -0.00072 | 0.00259 |  | - | - | - |
| Baseline | 0.01851 | | -0.02262 | 0.06093 |  | 0.02360 | -0.01814 | 0.06562 |  | **0.20939** | 0.16193 | 0.25696 |  | 0.00045 | -0.00072 | 0.00259 |  | - | - | - |
| Threat | 0.00178 | | -0.04118 | 0.04427 |  | 0.02185 | -0.02035 | 0.06450 |  | **0.14558** | 0.09703 | 0.19407 |  | 0.00004 | -0.00135 | 0.00162 |  | - | - | - |
| Parent Ed | 0.01407 | | -0.02787 | 0.05545 |  | 0.02392 | -0.01890 | 0.06761 |  | **0.20576** | 0.15865 | 0.25311 |  | 0.00035 | -0.00094 | 0.00242 |  | - | - | - |

*Note.* Bayesian mixed-effects regression models were used to estimate associations between adversity dimensions (threat and deprivation), pubertal timing, and mental health problems (internalizing and externalizing). Est, Estimate; CI L, lower bound credibility interval; CI U, upper bound credibility interval; MH, mental health; Primary, primary analysis model; Baseline, baseline mental health problems included as a covariate; Deprivation/Threat, alternate adversity factor included as a covariate; Parent Ed, mean parent education included as a covariate. *This direct effect was no longer significant when baseline internalzing and externalizing were added as covariates.

**Table S10**

Bayesian R-Squared Values for Primary Multivariate Regression Models

|  | Model A1 | | | |  | Model B1 + C’ | | | |
| --- | --- | --- | --- | --- | --- | --- | --- | --- | --- |
|  | B *R*^2^ | Error | 95% CI L | 95% CI U |  | B *R*^2^ | Error | 95% CI L | 95% CI U |
| Threat |  |  |  |  |  |  |  |  |  |
| Internalizing | 0.3291 | 0.0204 | 0.2886 | 0.3681 |  | 0.7935 | 0.0036 | 0.7863 | 0.8005 |
| Externalizing | 0.3302 | 0.0202 | 0.2906 | 0.3688 |  | 0.8040 | 0.0035 | 0.7969 | 0.8107 |
| Deprivation |  |  |  |  |  |  |  |  |  |
| Internalizing | 0.3286 | 0.0200 | 0.2889 | 0.3670 |  | 0.7946 | 0.0036 | 0.7873 | 0.8014 |
| Externalizing | 0.3284 | 0.0206 | 0.2670 | 0.3681 |  | 0.8035 | 0.0035 | 0.7965 | 0.8101 |

*Note.* B *R*^2^, Bayesian *R*^2^; CI L, lower bound credibility interval; CI U, upper bound credibility interval; Model A1, regression model for *a*1 pathway (pubertal_timing ~ adversity + covariates); Model B1 + C’, regresstion model for *b*1 and *c*’ pathways (mental_health_problems ~ adversity + pubertal_timing + covariates). Bayesian *R*^2^ estimated using the “bayes_R2” function from the *brms* package (Bürkner, 2017). It is calculated as the predicted variance divided by predicited variance plus error variance and provides a measure of variance explained in the outcome variable by the predictor variables in the regression model from the estimated values in the posterior (Gelman, Goodrich, Gabry, & Vehtari, 2019).

**Moderated Mediation**

**Figure S5**

*Moderated Mediation Model & Conditional Process Pathways of Interest*

*
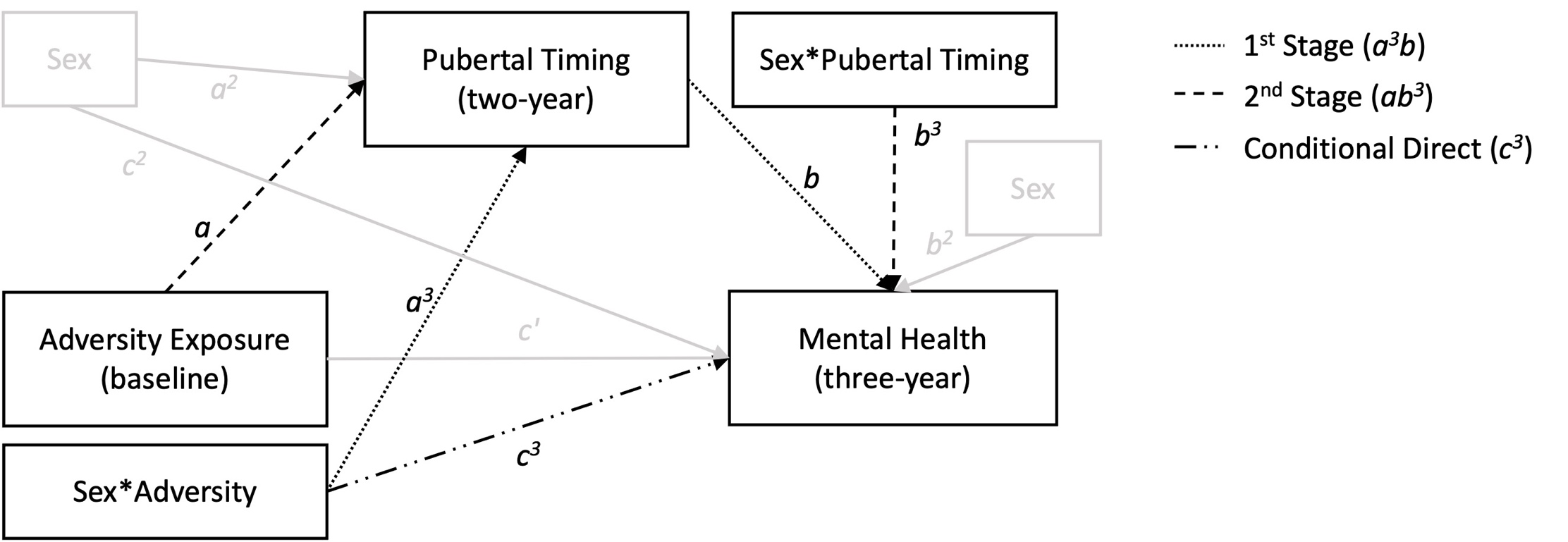
*

*Note.* Pathways of interest presented in black, non-examined pathways presented in grey. *, interaction term. 1^st^ Stage, moderating effect of sex on the association between adversity exposure and pubertal timing when pubertal timing mediates adversity and mental health problems; 2^nd^ Stage, moderating effect of sex on the association between pubertal timing and mental health problems when pubertal timing mediates adversity and mental health problems; Conditional Direct, moderating effect of sex on the association between adversity and mental health problems.

**Table S11**

*Conditional Effects of Sex on Adversity, Pubertal Timing, and Mental Health Problems*

|  | Threat-Internalizing | | |  | Threat-Externalizing | | |  | Deprivation-Internalizing | | |  | Deprivation-Externalizing | | |
| --- | --- | --- | --- | --- | --- | --- | --- | --- | --- | --- | --- | --- | --- | --- | --- |
|  | Est | 95%CI L | 95%CI U |  | Est | 95%CI L | 95%CI U |  | Est | 95%CI L | 95%CI U |  | Est | 95%CI L | 95%CI U |
| 1^st^ & 2^nd^ Stage | -0.00097 | -0.00609 | 0.00376 |  | -0.00038 | -0.00771 | 0.00557 |  | 0.00027 | -0.00631 | 0.00703 |  | 0.00144 | -0.00569 | 0.01006 |
| 1^st^ Stage | -0.00097 | -0.00609 | 0.00376 |  | -0.00034 | -0.00374 | 0.00222 |  | 0.00263 | -0.00030 | 0.00690 |  | 0.00128 | -0.00096 | 0.00490 |
| 2^nd^ Stage | 0.00131 | -0.00192 | 0.00540 |  | -0.00009 | -0.00445 | 0.00420 |  | 0.00071 | -0.00113 | 0.00318 |  | -0.00003 | -0.00244 | 0.00244 |
| Conditional Direct | **-0.15699** | **-0.24211** | **-0.07175** |  | 0.01844 | -0.08083 | 0.11610 |  | **-0.09808** | **-0.15660** | **-0.04026** |  | -0.02698 | -0.09594 | 0.04123 |
| Path a3 | -0.02044 | -0.11217 | 0.07352 |  | -0.01663 | -0.10929 | 0.07632 |  | 0.05473 | -0.00710 | 0.11537 |  | 0.05393 | -0.00836 | 0.11622 |
| Path b3 | 0.01496 | -0.02209 | 0.05178 |  | -0.00102 | -0.04481 | 0.04175 |  | 0.01433 | -0.02148 | 0.05105 |  | -0.00082 | -0.04429 | 0.04369 |

*Note.* Bayesian mixed-effects regression models were used to estimate associations between adversity dimensions (threat and deprivation), pubertal timing, and mental health problems (internalizing and externalizing). All presented values represent the difference between the specified pathway that has been estimated at each level of the moderator (sex). Est, Estimate; 95%CI L, lower bound credibility interval; 95%CI U, upper bound credibility interval; 1^st^ Stage, moderating effect of sex on the association between adversity exposure and pubertal timing in a model where pubertal timing mediates adversity and mental health problems; 2^nd^ Stage, moderating effect of sex on the association between pubertal timing and mental health problems in a model where pubertal timing mediates adversity and mental health problems; 1^st^ & 2^nd^ Stage, moderating effect of sex on the association between adversity exposure and pubertal timing as well as pubertal timing and mental health in a model where pubertal timing mediates adversity and mental health problems; Conditional Direct, moderating effect of sex on the association between adversity and mental health problems; a3, moderating effect of sex on the association of adversity and pubertal timing; b3, moderating effect of sex on the association of pubertal timing and mental health problems.

**Figure S6**

*Sex Differences in the Association Between Adversity Exposure and Pubertal Timing*

**
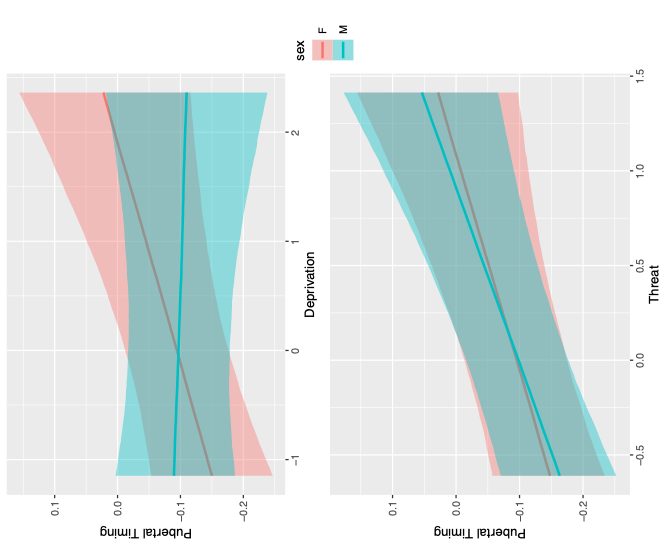
**

*Note.* Plotted data are results of a post-hoc exploratory linear regression that used the same variables inputed into the primary Bayesian regression analyses. Dimensional adversity exposure is plotted on the x-axis and pubertal timing on the y-axis. Effects for females represented in red and males in blue.

**Four-Factor Adversity Model Mediation**

Supplemental analyses were conducted to examine whether pubertal timing mediated the potential relationship between SES and household instability adversity exposures and mental health outcomes. As described, 2961 participants had missingness on baseline items that formed the SES and household instability adversity factors. It was found that participants with and without missingness on these items significantly differed on several demographic variables and PDS scores (Table S1). Accordingly, latent factor scores for participants with missingness of SES and household instability items were imputed so that mediation analysis could be conducted on the same sample of participants as the primary mediation models. Imputation and mediation methods are the same as those described for the primary analysis. Results of Bayesian mediation models are presented in Table S12. These showed that results for threat and deprivation were consistent with those found in the primary (two factor) analysis. Higher levels of exposure to both SES and household instability were associated with more internalizing and externalizing problems. Higher levels of exposure to these adversities were also associated with earlier pubertal timing. Pubertal timing mediated the association between SES and household instability exposure and internalizing problems. There were no indirect effects found for externalizing problems.

**Table S12**

*Four-Factor Mediation Models: Mediating Effects of Pubertal Timing on Adversity Exposure and Mental Health Problems*

|  | Adversity – Pubertal Timing | | |  | Pubertal Timing – MH Problems | | |  | Adversity – MH Problems | | |  | Indirect Effect of Pubertal Timing | | |
| --- | --- | --- | --- | --- | --- | --- | --- | --- | --- | --- | --- | --- | --- | --- | --- |
|  | Est | 95% CI L | 95% CI U |  | Est | 95% CI L | 95% CI U |  | Est | 95% CI L | 95% CI U |  | Est | 95% CI L | 95% CI U |
| Internalizing | |  |  |  |  |  |  |  |  |  |  |  |  |  |  |
| Threat | **0.12869** | 0.09292 | 0.16502 |  | **0.03768** | 0.00955 | 0.06599 |  | **0.39890** | 0.36281 | 0.43544 |  | **0.00486** | 0.00120 | 0.00917 |
| Deprivation | 0.02788 | -0.00609 | 0.06230 |  | **0.03797** | 0.00919 | 0.06686 |  | **0.12880** | 0.09486 | 0.16284 |  | 0.00107 | -0.00021 | 0.00300 |
| SES | **0.09094** | 0.06239 | 0.12016 |  | **0.03881** | 0.00941 | 0.06766 |  | **0.08505** | 0.05155 | 0.11791 |  | **0.00353** | 0.00080 | 0.00664 |
| Instability | **0.20282** | 0.15577 | 0.24938 |  | **0.04084** | 0.01221 | 0.06976 |  | **0.53075** | 0.48225 | 0.57978 |  | **0.00829** | 0.00235 | 0.01490 |
| Externalizing | |  |  |  |  |  |  |  |  |  |  |  |  |  |  |
| Threat | **0.12644** | 0.08996 | 0.16290 |  | 0.02225 | -0.00967 | 0.05416 |  | **0.50724** | 0.46378 | 0.55081 |  | 0.00275 | -0.00114 | 0.00717 |
| Deprivation | 0.02503 | -0.01009 | 0.06031 |  | 0.02065 | -0.01079 | 0.05287 |  | **0.23145** | 0.19173 | 0.26977 |  | 0.00053 | -0.00037 | 0.00213 |
| SES | **0.08989** | 0.06099 | 0.11924 |  | 0.01990 | -0.01289 | 0.05274 |  | **0.17606** | 0.13533 | 0.21778 |  | 0.00179 | -0.00112 | 0.00494 |
| Instability | **0.19981** | 0.15200 | 0.24770 |  | 0.02490 | -0.00673 | 0.05698 |  | **0.62066** | 0.56365 | 0.67959 |  | 0.00499 | -0.00133 | 0.01201 |

*Note.* MH, mental health; Est, Estimate; CI L, lower bound credibility interval; CI U, upper bound credibility interval. Each row represents an independent model.

**Supplementary References**

Bürkner, P.-C. (2017). brms: An R Package for Bayesian Multilevel Models Using Stan. *Journal of Statistical Software, 80*(1). doi:10.18637/jss.v080.i01

Buuren, S. v., & Groothuis-Oudshoorn, K. (2011). mice: Multivariate Imputation by Chained Equations inR. *Journal of Statistical Software, 45*(3). doi:10.18637/jss.v045.i03

Department of Health and Human Services. (January 18, 2018). *Annual Update of the HHS Poverty Guidelines 2018* (83 FR 2642). Retrieved from Federal Register: <https://www.federalregister.gov/d/2018-00814>

Gelman, A., Goodrich, B., Gabry, J., & Vehtari, A. (2019). R-squared for Bayesian Regression Models. *The American Statistician, 73*(3), 307-309. doi:10.1080/00031305.2018.1549100

Gelman, A., Simpson, D., & Betancourt, M. (2017). The Prior Can Often Only Be Understood in the Context of the Likelihood. *Entropy, 19*(10). doi:10.3390/e19100555

Hayes, A. F., & Rockwood, N. J. (2019). Conditional Process Analysis: Concepts, Computation, and Advances in the Modeling of the Contingencies of Mechanisms. *American Behavioral Scientist, 64*(1), 19-54. doi:10.1177/0002764219859633

Kim, K., Mennen, F. E., & Trickett, P. K. (2017). Patterns and correlates of co-occurrence among multiple types of child maltreatment. *Child & Family Social Work, 22*(1), 492-502. doi:10.1111/cfs.12268

R Core Team. (2021). R: A language and environment for statistical computing. Vienna, Austria: R Foundation for Statistical Computing. Retrieved from <https://www.R-project.org/>

Rosseel, Y. (2012). lavaan: An R Package for Structural Equation Modeling. *Journal of Statistical Software, 48*(2). doi:10.18637/jss.v048.i02

Team, R. (2020). RStudio: Integrated Development for R. Boston, MA: RStudio, PBC. Retrieved from <http://www.rstudio.com/>.

Vehtari, A. (2024). Prior Choice Recommendations. *Stan Development.* Retrieved from <https://github.com/stan-dev/stan/wiki/Prior-Choice-Recommendations>

Vehtari, A., Gelman, A., Simpson, D., Carpenter, B., & Bürkner, P.-C. (2021). Rank-Normalization, Folding, and Localization: An Improved Rˆ for Assessing Convergence of MCMC (with Discussion). *Bayesian Analysis, 16*(2). doi:10.1214/20-ba1221

Yuan, Y., & MacKinnon, D. P. (2009). Bayesian mediation analysis. *Psychological Methods, 14*(4), 301-322. doi:10.1037/a0016972
